# Ubiquitination contributes to the regulation of GDP-mannose pyrophosphorylase B activity

**DOI:** 10.3389/fnmol.2024.1375297

**Published:** 2024-06-24

**Authors:** Patricia Franzka, Sonnhild Mittag, Abhijnan Chakraborty, Otmar Huber, Christian A. Hübner

**Affiliations:** ^1^Institute of Human Genetics, Jena University Hospital, Friedrich Schiller University, Jena, Germany; ^2^Department of Biochemistry II, Jena University Hospital, Friedrich Schiller University, Jena, Germany

**Keywords:** GMPPB, ubiquitination, activity, Neuro-2a, glycosylation, TRIM67

## Abstract

GDP-mannose pyrophosphorylase B (GMPPB) loss-of-function is associated with muscular dystrophy and variable additional neurological symptoms. GMPPB facilitates the catalytic conversion of mannose-1-phosphate and GTP to GDP-mannose, which serves as a mannose donor for glycosylation. The activity of GMPPB is regulated by its non-catalytic paralogue GMPPA, which can bind GDP-mannose and interact with GMPPB, thereby acting as an allosteric feedback inhibitor of GMPPB. Using pulldown, immunoprecipitation, turnover experiments as well as immunolabeling and enzyme activity assays, we provide first direct evidence that GMPPB activity is regulated by ubiquitination. We further show that the E3 ubiquitin ligase TRIM67 interacts with GMPPB and that knockdown of TRM67 reduces ubiquitination of GMPPB, thus reflecting a candidate E3 ligase for the ubiquitination of GMPPB. While the inhibition of GMPPB ubiquitination decreases its enzymatic activity, its ubiquitination neither affects its interaction with GMPPA nor its turnover. Taken together, we show that the ubiquitination of GMPPB represents another level of regulation of GDP-mannose supply.

## Introduction

GDP-mannose pyrophosphorylase B (GMPPB) catalyzes the conversion of guanosine triphosphate and mannose-1-phosphate to GDP-mannose, which serves as the activated form of mannose required for glycosylation. This complex process is initiated in the endoplasmic reticulum (ER) and proceeds in the Golgi apparatus. Glycosylation can have important consequences for protein folding, stability, localization, turnover, and protein-protein interactions (Varki et al., 2009). For example, hypoglycosylation due to decreased GMPPB activity decreases the stability and abundance of α-dystroglycan (Franzka et al., 2021a). α-dystroglycan is a peripheral membrane component of the dystrophin-glycoprotein complex (DGC), which can be found in the muscle, nerve, heart, and brain (Ervasti and Campbell, 1991; Carss et al., 2013; Belaya et al., 2015; Jensen et al., 2015; Astrea et al., 2018). Mutations in GMPPB lead to variable neurological diseases ranging from severe congenital muscular dystrophy to myasthenic syndromes with eye and brain symptoms (Carss et al., 2013; Belaya et al., 2015). Notably, disease severity correlates with residual GMPPB activity (Liu et al., 2021).

GDP-mannose pyrophosphorylase A (GMPPA) is a catalytically inactive paralogue of GMPPB, which can still bind GDP-mannose. We have previously shown that GMPPA directly interacts with GMPPB and inhibits the activity of GMPPB in a GDP-mannose-dependent manner (Franzka et al., 2021a). Thus, the absence of GMPPA in men (Koehler et al., 2013) or mice (Franzka et al., 2021a) results in increased GDP-mannose levels, hypermannosylation of different proteins, including α-dystroglycan, and characteristic neurological symptoms (Franzka et al., 2021a).

Notably, GMPPB itself is also a substrate for post-translational modifications, including glycosylation, phosphorylation, and ubiquitination (Hornbeck et al., 2015). Ubiquitination refers to the covalent binding of ubiquitin at lysine residues of the target protein, which involves the concerted action of E1 ubiquitin-activating enzymes, E2 ubiquitin-conjugating enzymes, and E3 ubiquitin-protein ligases (Hershko and Ciechanover, 1998; Dikic and Schulman, 2023). The latter constitutes a large superfamily with three main subclasses: Homologous to E6AP C-Terminus (HECT) E3 ubiquitin ligases, Really Interesting New Gene/U-box (RING) E3 ubiquitin ligases, and RING between RING (RBR) E3 ubiquitin ligases (Uchida and Kitagawa, 2016; Yang et al., 2021). Ubiquitination occurs in various forms, ranging from simple mono-ubiquitination to polymeric ubiquitin chains with complex topologies (Kwon and Ciechanover, 2017).

One of the best-understood functions of ubiquitination is protein degradation, which is achieved by targeting proteins to the proteasome (Kaiser and Huang, 2005; Kirkpatrick et al., 2006). Ubiquitin tagging can also provide a signal to target proteins for lysosomal degradation (Komander and Rape, 2012). However, ubiquitination can also act as a regulatory signal that alters the activity, localization, and ultimate fate of the respective protein. For example, we recently showed that ubiquitination promotes the clustering of ER membrane-shaping proteins and its binding to LC3, thus regulating the degradation of ER-fragments by ER-phagy (Khaminets et al., 2015; Foronda et al., 2023; González et al., 2023). Ubiquitination can also promote the interaction between proteins and even regulate enzyme activities (Walczak et al., 2012; Swatek and Komander, 2016; Wang et al., 2022).

In this study, we provide the first evidence that the enzymatic activity of GMPPB is modulated by ubiquitination, revealing an additional level of regulation in addition to allosteric feedback inhibition via GMPPA.

## Methods

### Cell culture

All cell lines used in this study were obtained from ATCC, Wesel (Germany). HEK-293 T, HEK-293, and Neuro-2a (N2A) cells were cultured in DMEM Glutamax (Sigma-Aldrich, Sant Gallen, Switzerland) supplemented with 10% (v/v) of FBS (Gibco, Dreieich, Germany) and 1% (v/v) of penicillin/streptomycin (Gibco, Dreieich, Germany) at 37°C.

### MG132, bafilomycin A1, and cycloheximide treatment

HEK-293 T cells were seeded and treated with either 10 μM of MG132 (Merck, Darmstadt, Germany) or 100 nM of bafilomycin A1 (Merck, Darmstadt, Germany) for different time points. Then, cells were lysed in lysis buffer (50 mM of Tris–HCl pH 7.4, 150 mM of NaCl, 1% (v/v) of NP-40, 1% (w/v) of sodium deoxycholate, 0.1% (w/v) of SDS, and 1 mM of EDTA) and complete protease inhibitor (Roche, Mannheim, Germany). Homogenates were centrifuged at 16,900 *g* to remove nuclei and insoluble debris. Protein concentration was determined using the Pierce BCA assay kit (Thermo Fischer, Dreieich, Germany). The supernatant was stored at −80°C until further use. For degradation analysis of overexpressed GMPPB WT and mutant constructs, HEK-293 T cells were transfected with vectors encoding 3xFLAG-GMPPB, 3xFLAG-GMPPB D334N, and 3xFLAG-GMPPB K143/162/195R with lipofectamine 200 reagent (Invitrogen, Dreieich, Germany). The next day, cells were treated with 100 nM of bafilomycin (Merck, Darmstadt, Germany) for 15 h. Then, the cells were lysed and processed as described above. For turnover experiments, HEK-293 T cells were either transfected with vectors encoding 3xFLAG-GMPPB, 3xFLAG-GMPPB D334N, or 3xFLAG-GMPPB K143/162/195R with lipofectamine 200 reagent (Invitrogen, Dreieich, Germany). The next day, cells were treated with 10 μM of cycloheximide (Sigma-Aldrich, Sant Gallen, Switzerland) at different time points. Then, cells were lysed and processed as described above.

### Co-localization and immunofluorescence microscopy

N2A cells were seeded, either not transfected or transfected with the vector encoding 3xFLAG-GMPPB WT using the lipofectamine 2000 reagent (Invitrogen, Dreieich, Germany), and treated with Earle’s Balanced Salt Solution (EBSS, Thermo Fischer, Dreieich, Germany) containing 1% (v/v) of penicillin/streptomycin and/or 100 nM of bafilomycin (Merck, Darmstadt, Germany) for 6 h. Cells were fixed with 4% of paraformaldehyde (PFA), blocked with 5% (v/v) with normal goat serum, and incubated with primary antibodies overnight at 4°C. For immunofluorescence detection, the following primary antibodies were used: rabbit anti-GMPPB (Proteintech, 15,094-1-AP, Planegg-Martinsried, Germany) 1:100, mouse anti-p62 (Abcam, ab56416, Cambridge, United Kingdom) 1:500, rat anti-LAMP1 (BD Pharmingen, 553,792, Heidelberg, Germany) 1:500, and rabbit anti-FLAG (Sigma-Aldrich, F7425) 1:1000. Corresponding secondary antibodies were obtained from Invitrogen. Nuclei were stained with DAPI 1:10,000 (Thermo Fischer, H3569, Dreieich, Germany). Samples were mounted with Fluoromount-G (Southern Biotech, Eching, Germany). Images were taken using a Zeiss LSM880, Jena (Germany) Airyscan confocal microscope. Z-projections with average intensities processed with ImageJ are shown. Co-localization was analyzed with the Comdet v05 plugin from ImageJ.

For localization analysis of overexpressed WT and mutant GMPPB protein, HEK-293 T cells were either transfected with 3xFLAG-GMPPB WT, 3xFLAG-GMPPB D334N, or 3xFLAG-GMPPB K143/162/195R with lipofectamine 2000 (Invitrogen, Dreieich, Germany). The next day, cells were fixed with 4% of PFA, blocked with 5% (v/v) of normal goat serum, and incubated with rabbit anti-FLAG M2 antibody (Sigma-Aldrich, F3165, Sant Gallen, Switzerland) 1:1,000 overnight at 4°C. After washing with phosphate-buffered saline (PBS) buffer, specimens were incubated with the respective secondary antibodies (Invitrogen, Dreieich, Germany) for 2 h and subsequently treated with a mounting medium. Imaging was performed using a Keyence BZ-X800E, Erfurt (Germany) microscope.

### Co-immunoprecipitation and Ni-NTA pulldown

HEK-293 T or HEK-293 cells were either transfected with vectors encoding GMPPA-Myc_6_, 3xFLAG-GMPPB, 3xFLAG-GMPPB D27H, 3xFLAG-GMPPB P103L, 3xFLAG-GMPPB R287Q, 3xFLAG-GMPPB D334N, 3xFLAG-GMPPB K143/162/195R, His_6_-ubiquitin or HA-ubiquitin, or HA-TRIM67 (the TRIM67 cDNA was a gift from Sevin Turcan (DKFZ Heidelberg, Germany)), using lipofectamine 2000 reagent (Invitrogen, Dreieich, Germany) or polyethylenimine. For the knockdown of TRIM67, cells were transfected with 600 pmol of siRNA either directed against TRIM67 (Thermo Fischer, Dreieich, Germany) or scrambled control (Dharmacon, Colorado, Unites States) using the lipofectamine 2000 reagent (Invitrogen, Dreieich, Germany) a day before transfection with the respective vectors. Two days after transfection, cells were lysed using imidazole lysis buffer (20 mM of imidazole pH 8.0, 150 mM of NaCl, 2 mM of MgCl_2_, 300 mM of sucrose, and 0.25% (v/v) of Triton X-100) and centrifuged at 16,900 *g*. The supernatant was incubated with either anti-Myc- and anti-FLAG-antibodies coupled to Protein A Sepharose™ CL-4B (Cytiva, Glattbrugg, Switzerland) or Ni-NTA agarose (Qiagen, Hilden, Germany) at 4°C for 2 h. After 3–6 washing cycles with lysis buffer, samples were boiled at 95°C for 10 min in Laemmli sample buffer.

### Western blot analysis

Proteins were denatured at 95°C for 5 min in Laemmli buffer. After separation by SDS-PAGE (10% of polyacrylamide gels), proteins were transferred onto PVDF membranes (Whatman, Hilden, Germany, Roth, Dautphetal, Germany). Membranes were blocked in 2% (w/v) of bovine serum albumin (BSA) and incubated with primary antibodies at appropriate dilutions overnight at 4°C. The following primary antibodies were used: rabbit anti-GMPPA (Proteintech, 15,517-1-AP, Planegg-Martinsried, Germany) 1:500, rabbit anti-GMPPB (Proteintech, 15,094-1-AP, Planegg-Martinsried, Germany) 1:500, rabbit anti-GAPDH (Proteintech, 10,494-1-AP, Planegg-Martinsried, Germany) 1:1,000, rabbit anti-Myc (Merck, 06–340, Darmstadt, Germany) 1:1,000, rabbit anti-FLAG M2 (Sigma-Aldrich, F3165, Sant Gallen, Switzerland) 1:1,000, rabbit anti-ubiquitin (Proteintech, 10,201-2-AP, Planegg-Martinsried, Germany) 1:1,000, and rat anti-HA (Roche, 11,867,423,001, Mannheim, Germany) 1:1,000. Primary antibodies were detected with HRP-conjugated secondary antibodies. The detection was performed using the Clarity Western ECL Substrate Kit (BioRad). The quantification of bands was performed with ImageJ.

Coomassie blue staining of PVDF membranes was performed as described before (Franzka et al., 2021b) after protein detection. For Coomassie blue staining of transferred proteins, PVDF membranes were fixed for 3 min (10% (v/v) of acetic acid and 40% (v/v) of EtOH), stained in Coomassie blue solution (0.1% (w/v) of Brilliant Blue R (Serva), 45% (v/v) of EtOH, and 10% (v/v) of acetic acid) for 5 min, destained (10% (v/v) of acetic acid, 20% (v/v) of EtOH), rinsed in H_2_O, and then imaged.

### Enzyme activity assays

HEK-293 T cells were transfected with vectors either encoding 3xFLAG-GMPPB WT, 3xFLAG-GMPPB D334N, 3xFLAG-GMPPB K143/162/195R, or 3xFLAG-GMPPB K143/162/195R D334N with lipofectamine 2000 (Invitrogen, Dreieich, Germany). After 24 h, cells were treated with or without PYR-41 (Merck, Darmstadt, Germany) to inhibit ubiquitination. After another 24 h, proteins were isolated and enriched via methanol/chloroform precipitation for protein quantification. Protein pellets were homogenized in assay buffer (25 mM of Tris–HCl pH 7.5, 150 mM of NaCl, 4 mM of MgCl_2_, 0.01% (v/v) of Tween 20, 1 mM of dithiothreitol in ultrapure water supplemented with complete protein inhibitor cocktail (Roche, Mannheim, Germany)), and protein concentration was measured using the BCA assay kit (Thermo Fischer, Dreieich, Germany).

Recombinant human MBP-GMPPB and MBP-GMPPB K143/162/195R were expressed in *E. coli* (Merck, Darmstadt, Germany) using the pMal-c2X plasmids and purified on amylose-resin (New England Biolabs, Frankfurt, Germany).

The GDP-mannose-pyrophosphorylase activity was measured as described before (Franzka et al., 2021a) by determining the quantity of inorganic phosphate generated from pyrophosphate in the presence of mannose-1-phosphate (150 μM), GTP (300 μM), and excess pyrophosphatase (1 U/mL, Merck, Darmstadt, Germany) in assay buffer at 37°C for 60 min. Biochemical reactions were terminated by adding equal volumes of revelation buffer (0.03% (w/v) of malachite green (Sigma-Aldrich, Sant Gallen, Switzerland), 0.2% (w/v) of ammonium molybdate, 0.05% (v/v) of Triton X-100 in 0.7 M HCl) at 30°C for 5 min, and the absorbance was measured at 650 nm.

### Statistical analyses

For statistical analysis, raw data were analyzed for normal distribution with the Kolmogorov–Smirnov goodness-of-fit test or with graphical analysis using the Box-Plot and QQ-Plot. If appropriate, we either used one-way ANOVA, two-way ANOVA, or Student’s t-test. * indicates *p* < 0.01, ***p < 0.001 and ****p < 0.0001. For statistical analysis, we used GraphPad Prism 5. For all data, means with the standard error of the mean (SEM) or individual data points with SEM were shown.

## Results

### GMPPB is ubiquitinated

**Figure 1:**
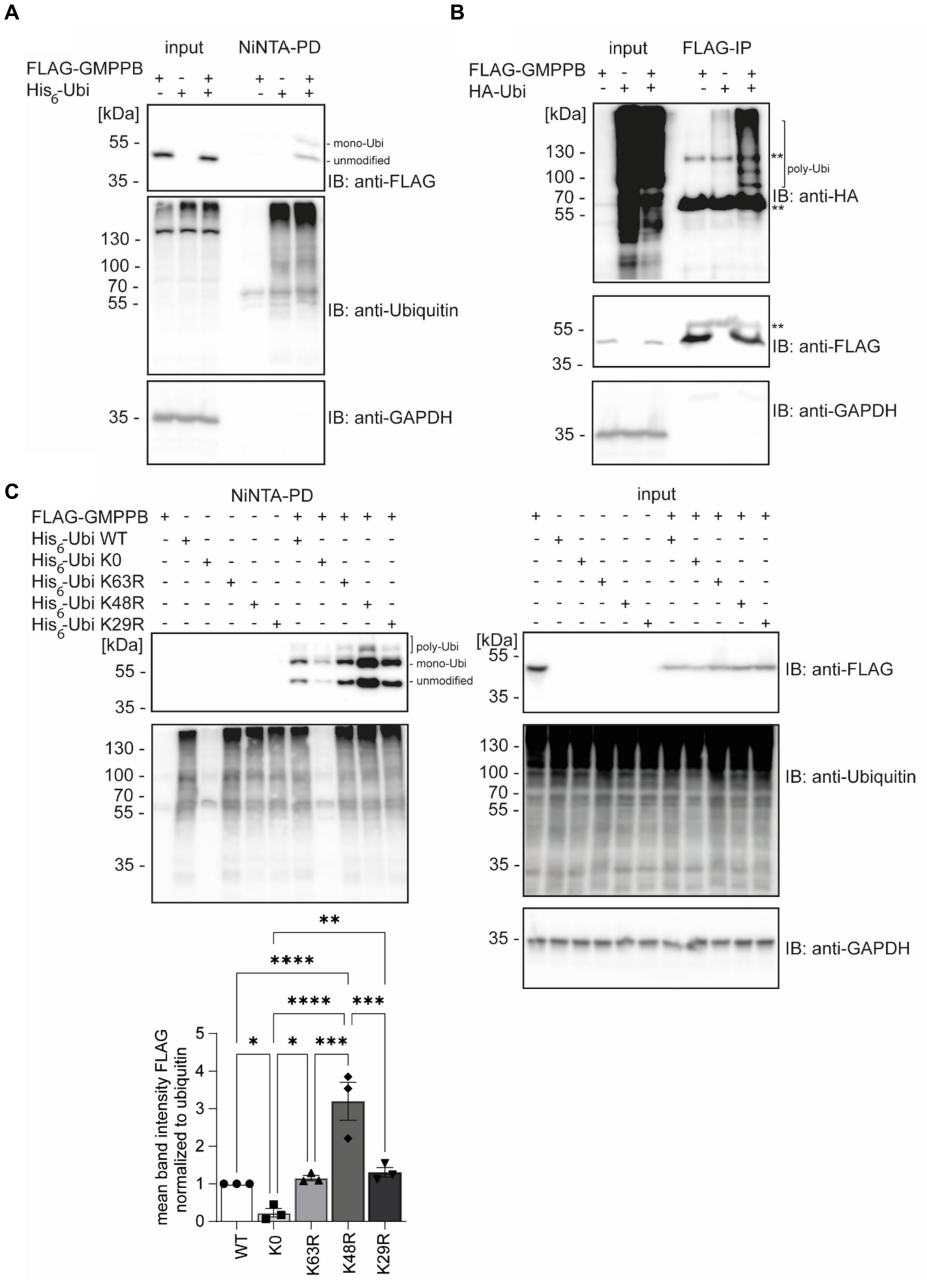
GMPPB is ubiquitinated. **(A)** Ubiquitinated GMPPB is pulled down with Ni-NTA beads from lysates of HEK-293 T cells, transiently overexpressing His_6_-ubiquitin and FLAG_3_-GMPPB. Input boxes represent 5% of the input used for pulldown. Unmodified and mono-ubiquitinated GMPPB bands (mono-Ubi) are indicated. **(B)** Ubiquitination is also evident upon overexpression of HA-ubiquitin and FLAG_3_-GMPPB and subsequent immunoprecipitation with anti-FLAG M2 antibodies. **heavy chain of the precipitating antibody. Poly-Ubiquitin bands (poly-Ubi) are indicated. Input boxes represent 5% of the input used for immunoprecipitation. **(C)** Ni-NTA pulldown experiments from cell lysates overexpressing FLAG_3_-GMPPB and His_6_-ubiquitin WT or His_6_-ubiquitin mutant constructs (K0, K29R, K48R, and K63R) show significantly reduced ubiquitination of GMPPB with the ubiquitin mutant K0, while GMPPB ubiquitination is increased with the ubiquitin mutant K48R compared to ubiquitin WT (*n* = 3 experiments, 1-way-ANOVA with Fischer’s LSD test). Unmodified, mono-ubiquitinated (mono-Ubi), and poly-ubiquitinated (poly-Ubi) GMPPB bands are indicated. Input boxes represent 5% of the input used for NiNTA-pulldown. Quantitative data are presented as mean ± SEM with individual data points. *p < 0.05; **p < 0.01; ***p < 0.001; ****p < 0.0001.

According to the PhosphoSitePlus database, lysines 143, 162, and 195 of GMPPB are reported to be ubiquitinated (Hornbeck et al., 2015; Akimov et al., 2018). To confirm that GMPPB is indeed a target for ubiquitination, we co-transfected HEK-293 T cells with FLAG-GMPPB and His_6_-ubiquitin constructs. Subsequently, ubiquitin and ubiquitinated proteins were pulled down from cell lysates with Ni-NTA beads and analyzed by Western blotting. Indeed, bands for ubiquitinated FLAG-tagged GMPPB were detectable in the pulldown (Figure 1A). Notably, upon co-precipitation of GMPPB with ubiquitin, a second higher molecular weight band of around 60 kDa was present, which was not visible in input samples that contained 5% of the input used for precipitation (Figure 1A). This higher molecular weight band potentially represents mono- and poly/multi-ubiquitinated GMPPB. To further confirm this assumption, we co-transfected HEK-293 T cells with FLAG-GMPPB and HA-ubiquitin constructs and immunoprecipitated GMPPB with anti-FLAG antibodies. Subsequent SDS-PAGE and Western blotting with an anti-HA-tag antibody revealed a ladder of ubiquitinated bands in co-transfected cells but not in cells transfected with HA-ubiquitin or FLAG-GMPPB alone (Figure 1B).

Next, we co-transfected HEK-293 T cells with FLAG-GMPPB and different ubiquitin variants (K0, K63R, K48R, and K29R). Again, we detected bands for FLAG-GMPPB at 50, 60, and 70 kDa, confirming the previous results (Figure 1C). In the ubiquitin K0 variant, all seven lysines were replaced by arginine, thus only allowing the ligation to the substrate as mono-ubiquitin. When we co-transfected the FLAG-GMPPB with the K0 construct, only weak signals for ubiquitinated GMPPB were detectable after pulldown with Ni-NTA agarose (Figure 1C, lane 8). The presence of two bands for ubiquitin K0-modified GMPPB suggests that GMPPB is ubiquitinated at more than one site. However, for unknown reasons, the enrichment of ubiquitin K0 was not as efficient as compared to WT ubiquitin variants (Figure 1C). We also assessed constructs, in which K29, K48, or K63 of ubiquitin were substituted by arginine, thus precluding the formation of the respective poly-ubiquitin chains. Although we did not observe a major difference upon co-transfection with either the ubiquitin K29R or K63R construct compared to ubiquitin WT (Figure 1C), the ubiquitination of GMPPB was significantly increased upon co-transfection of FLAG-GMPPB with the ubiquitin K48R variant. This suggests that either K48 is obstructive for poly-ubiquitination of GMPPB or that mono- or multi-ubiquitination of GMPPB via ubiquitin K48 leads to either stabilization or destabilization of the protein. Alternatively, the increase in ubiquitinated GMPPB upon co-transfection of FLAG-GMPPB with the WT ubiquitin may trigger proteasomal degradation, which is impaired when K48-poly-ubiquitination is prevented, thus leading to the accumulation of the protein.

Overall, our findings suggest that GMPPB is indeed ubiquitinated.

### Patient’s mutations can alter GMPPB ubiquitination

**Figure 2:**
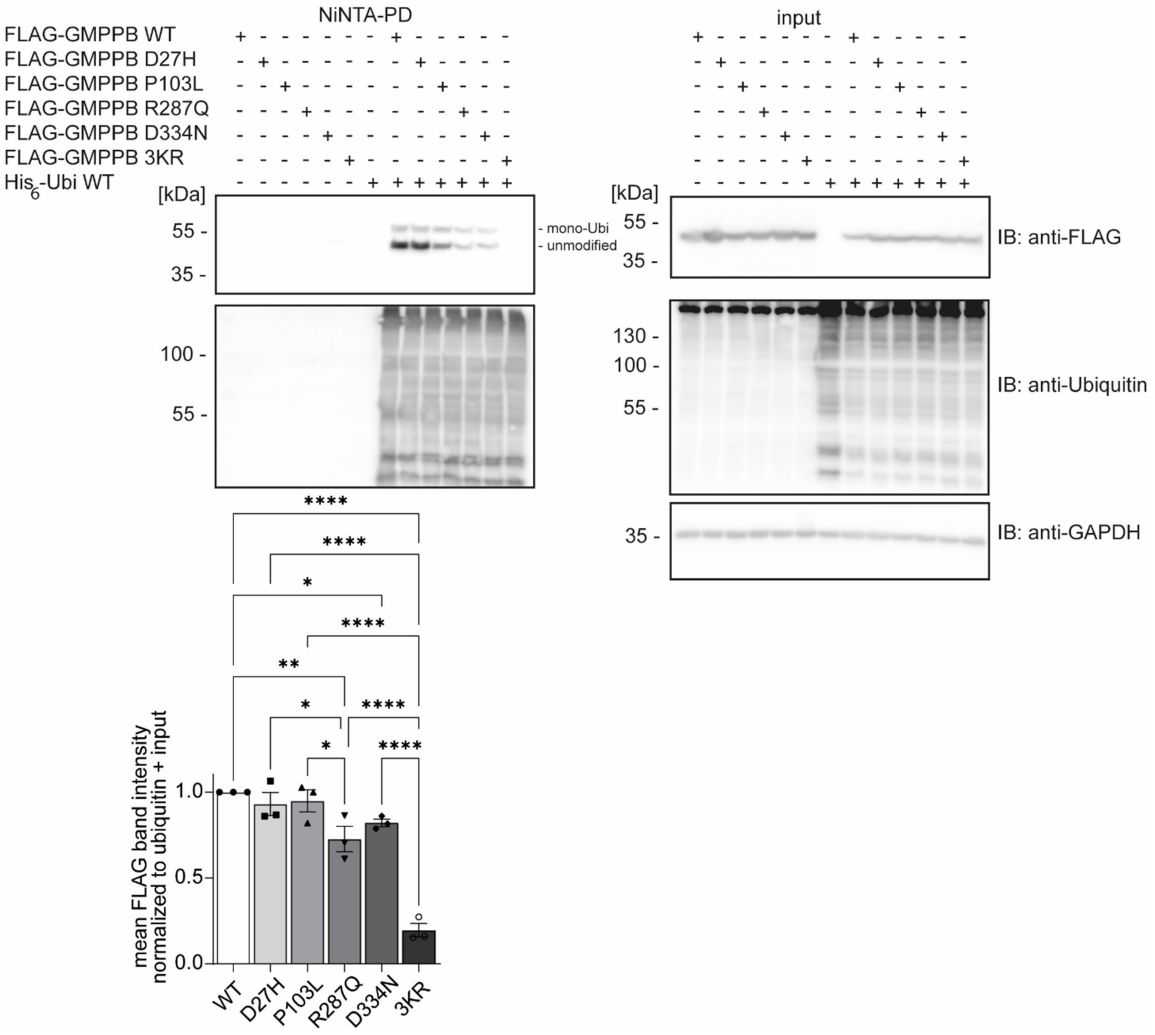
Patient mutations can alter the ubiquitination of GMPPB. Representative blots of Ni-NTA pulldown of overexpressed His_6_-ubiquitin and FLAG_3_-GMPPB WT and mutant constructs (D27H, P103L, R287Q, D334N, and K143/162/195R) and quantification (*n* = 3 experiments, 1-way-ANOVA with Fischer’s LSD test). GAPDH is the loading control for input samples. Intensities of pulled-down FLAG-GMPPB bands are normalized to the intensities of pulled-down ubiquitin bands and FLAG_3_-GMPPB bands detected in the input control (normalized to GAPDH). Unmodified and mono-ubiquitinated GMPPB bands (mono-Ubi) are indicated. Input boxes represent 5% of the input used for pulldown. Quantitative data are presented as mean ± SEM with individual data points. *p < 0.05; **p < 0.01; ***p < 0.001; ****p < 0.0001.

Disease-associated GMPPB variants include missense, nonsense, and frameshift mutations, which are assumed to result in GMPPB loss-of-function (Astrea et al., 2018; Tian et al., 2019). GMPPB activity is more severely compromised by missense mutations located in its N-terminal nucleotidyl-transferase domain compared to its C-terminal β-helix domain (Liu et al., 2021). The latter, however, often alters the subcellular localization of GMPPB (Carss et al., 2013; Belaya et al., 2015; Tian et al., 2019) or decreases the overall abundance of GMPPB (Belaya et al., 2015; Tian et al., 2019). We speculated whether the position of the respective patient variant might affect the ubiquitination of GMPPB. To this end, we analyzed the ubiquitination of the disease-associated variants D27H and P103L, which are located in the N-terminal part of GMPPB, as well as R287Q and D334N, which are located in the C-terminal part of GMPPB, in HEK-293 T cells (Figure 2). In addition, we cloned a construct in which the three ubiquitination sites K143, K162, and K195 were replaced by arginine (GMPPB 3KR) (Figure 2). Notably, we could not detect FLAG-GMPPB 3KR upon Ni-NTA pulldown of His_6_-ubiquitin (Figure 2). GMPPB mutants R287Q and D334N harboring patient mutations in the C-terminal part of the protein showed reduced ubiquitination (Figure 2), while GMPPB mutants with patient mutations in its N-terminal part were not affected (Figure 2).

Overall, these data suggest that patient mutations located in the C-terminal part of GMPPB may influence the ubiquitination status of GMPPB.

### GMPPB interacts with the E3 ubiquitin ligase TRIM67

**Figure 3:**
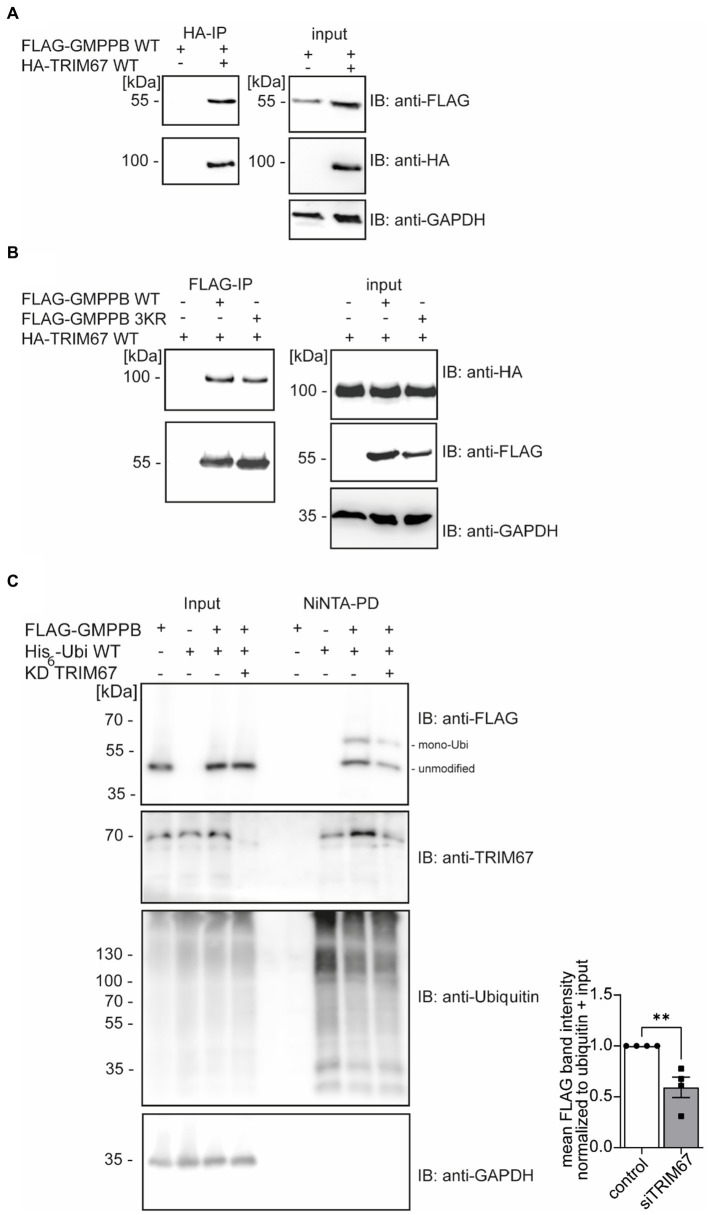
GMPPB interacts with the E3 ubiquitin ligase TRIM67. **(A)** Overexpressed FLAG_3_-GMPPB co-precipitates with HA-TRIM67. GAPDH is the loading control for the input. Input boxes represent 5% of the input used for precipitation. **(B)** Overexpressed HA-TRIM67 co-precipitates with FLAG_3_-GMPPB WT and FLAG_3_-GMPPB 3KR (K143R, K162R, and K195R) upon IP with anti-FLAG M2 antibodies. GAPDH is the loading control for input samples. Input boxes represent 5% of the input used for precipitation. **(C)** Representative immunoblots of Ni-NTA pulldown of overexpressed His_6_-ubiquitin and FLAG_3_-GMPPB WT at control condition (scrambled siRNA) or upon siRNA mediated knockdown and quantification (*n* = 4 experiments, Student’s t-Test). GAPDH is the loading control for input samples. The Intensities of pulled-down FLAG_3_-GMPPB bands are normalized to the intensity of pulled-down ubiquitin bands and FLAG_3_-GMPPB bands detected in the input control (normalized to GAPDH). Unmodified and mono-ubiquitinated GMPPB bands (mono-Ubi) are indicated. Input boxes represent 5% of the input used for precipitation. Quantitative data are presented as mean ± SEM with individual data points. **p < 0.01.

In the biogrid database, we found that the RING E3 ligase TRIM67 is predicted to interact with GMPPB (Oughtred et al., 2021; Demirdizen et al., 2023). To verify the GMPPB interaction with TRIM67, we transfected HEK-293 T cells with FLAG_3_-GMPPB and HA-TRIM67 constructs, respectively. Indeed, upon immunoprecipitation of HA-TRIM67, a band for FLAG-tagged GMPPB was clearly visible (Figure 3A). To further confirm this result, we co-transfected HEK-293 T cells with the constructs for HA-TRIM67 and FLAG_3_-GMPPB WT and the mutant devoid of all three reported ubiquitination sites (K143/162/195R: GMPPB 3KR) and immunoprecipitated GMPPB WT and variant protein with anti-FLAG M2 antibodies. On Western blots, bands for HA-TRIM67 were visible when GMPPB WT and mutated constructs were present (Figure 3B). To further validate this finding, we knocked down TRIM67 in HEK-293 T cells before co-transfection of FLAG_3_-GMPPB and His_6_-ubiquitin. Upon Ni-NTA pulldown of His_6_-ubiquitin, approximately 50% less ubiquitination of GMPPB was visible upon TRIM67 knockdown (Figure 3C).

In summary, these data show that the ubiquitination of GMPPB is at least in part mediated by TRIM67.

### GMPPB abundance is controlled by autophagy

**Figure 4:**
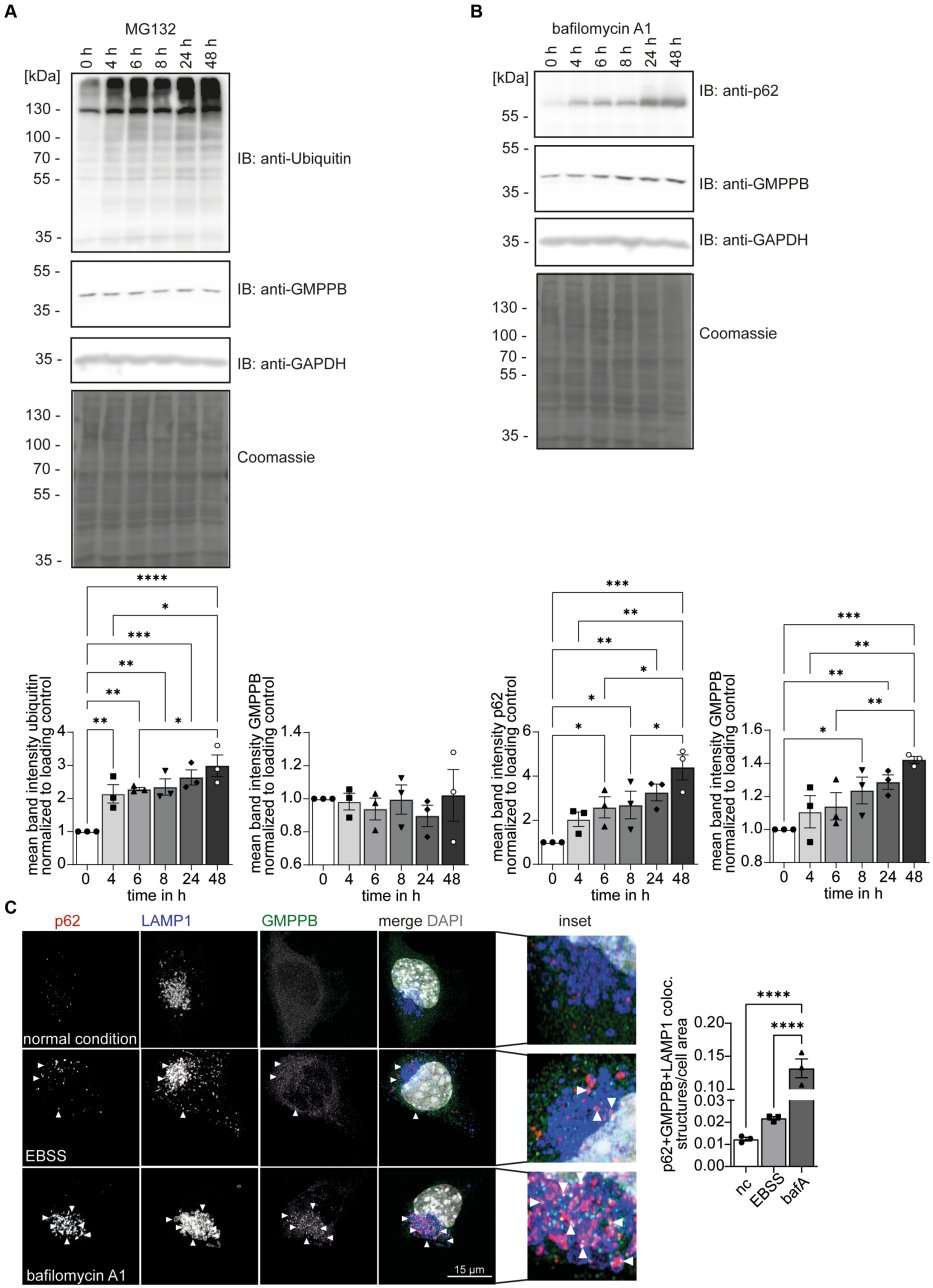
GMPPB protein levels are controlled via autophagy. **(A)** The inhibition of proteasomal protein degradation with MG132 does not affect the abundance of endogenous GMPPB in HEK-293 T cells. Detection of ubiquitin served as a control for efficient MG132 treatment. GAPDH and Coomassie staining are the loading control. Mean band intensities are normalized to GAPDH (*n* = 3 experiments, 1-way-ANOVA with Fischer’s LSD test). **(B)** The inhibition of lysosomal degradation with bafilomycin A1 in HEK-293 T cells leads to an increase in the abundance of GMPPB. The accumulation of SQSTM1/p62 in the presence of bafilomycin A1 confirms the inhibition of lysosomal degradation. GAPDH and Coomassie staining served as loading control. Mean band intensities are normalized to GAPDH (*n* = 3 experiments, 1-way-ANOVA with Fischer’s LSD test). **(C)** EBSS starvation for 6 h or the inhibition of lysosomal degradation with bafilomycin A1 increases the co-localization of GMPPB with autolysosomes (LAMP1- and p62-positive puncta) in N2A cells. Co-localization is analyzed with the Comdet v05 plugin from ImageJ. *N* = 3 experiments with 10–15 cells/genotype per condition and experiment, scale bar: 15 μm. White arrowheads indicate exemplary co-localization. Quantitative data are presented as mean ± SEM with individual data points. *p < 0.05; **p < 0.01; ***p < 0.001; ****p < 0.0001.

Ubiquitination can target proteins for proteasomal (Kaiser and Huang, 2005; Kirkpatrick et al., 2006) or lysosomal degradations (Marques et al., 2004; Clague and Urbé, 2010). To test whether ubiquitination controls the turnover of GMPPB, we treated HEK-293 T cells with either MG132 to block proteasomal function or bafilomycin A1 to block lysosomal degradation. Increasing ubiquitin levels confirmed that MG132 treatment was effective (Figure 4A). The inhibition of lysosomal degradation by bafilomycin A1 was confirmed by the increase in the abundance of SQSTM1/p62 (Figure 4B). Because SQSTM1/p62 targets proteins in autophagosomes for degradation and is degraded itself during autophagy, increasing SQSTM1/p62 levels reflect efficient inhibition of lysosomal degradation by bafilomycin A1. While the abundance of GMPPB did not change by inhibiting proteasomal degradation (Figure 4A), the accumulation of GMPPB upon bafilomycin A1 treatment suggested that GMPPB is degraded by the lysosomal pathway (Figure 4B).

There are two major lysosomal-based degradation pathways: the degradation of endocytosed proteins by the endosomal pathway and the degradation of proteins and damaged organelles by autophagy. To further study the role of autophagy for GMPPB turnover, we starved neuroblastoma-derived N2A cells (Evangelopoulos et al., 2005) for 6 h with Earle’s Balanced Salt Solution (EBSS) to induce autophagy by cell starvation (Figure 4C; Supplementary Figure S1B): Bafilomycin A1 treatment served as positive control for inhibition of autophagy. Immunofluorescence microscopic analysis for GMPPB, SQSTM1/p62, a marker for autophagosomes, and the lysosomal marker protein, LAMP1, allowed us to assess the co-localization of GMPPB with autolysosomes (p62- and LAMP1-positive structures) under normal conditions and upon induction of autophagy and upon inhibition of autophagy. Notably, EBSS treatment increased the co-localization of GMPPB with autolysosomes (p62- and LAMP1-positive), which drastically increased upon simultaneous inhibition of lysosomal degradation with bafilomycin A1 (Figure 4C; Supplementary Figure S1B). To further validate this finding, we overexpressed FLAG-tagged GMPPB in N2A cells and either induced autophagy by EBSS treatment for 6 h or blocked autophagy simultaneously with bafilomycin A1. Co-labeling of LAMP1, p62, and FLAG-tagged of GMPPB allowed us to quantify the co-localization of GMPPB with LAMP1 and p62. In agreement with Figure 4C, EBSS treatment increased the co-localization of GMPPB with autolysosomes, which was further triggered with bafilomycin A1 treatment (Supplementary Figures S1A,B). These data suggest that GMPPB is predominantly degraded via autophagy.

To assess whether the ubiquitination of GMPPB is necessary for its lysosomal degradation, we transfected HEK-293 T cells with a construct either encoding GMPPB WT, the disease-associated point-mutation GMPPB D334N, or GMPPB 3KR and blocked autophagy with bafilomycin A1 (Supplementary Figure S2A). Notably, the abundance of overexpressed WT and mutant GMPPB increased upon bafilomycin A1 treatment (Supplementary Figure S2A).

To assess whether the ubiquitination of GMPPB is important for its protein stability and turnover, we overexpressed GMPPB WT, the disease-associated variant GMPPB D334N or GMPPB 3KR in HEK-293 T cells, and blocked protein translation by cycloheximide (CHX). Decreasing ubiquitin levels confirmed an efficient inhibition of protein translation (Supplementary Figure S2B). GMPPB WT, D334N, and 3KR protein levels decreased over time in a similar ratio (Supplementary Figure S2B).

To assess if overexpression of GMPPB results in an accumulation within vesicular structures, such as lysosomes, we overexpressed GMPPB WT as well as mutant constructs in HEK-293T cells in the absence of bafilomycin A1 or EBSS. Cells transfected with GMPPB WT and GMPPB 3KR mutant showed a diffuse cytoplasmic staining pattern for GMPPB, whereas cells transfected with the GMPPB D334N mutant showed a more aggregated staining pattern (Supplementary Figure S2C). Overall, ubiquitination does not seem to regulate the degradation of GMPPB.

### The interaction of GMPPB and GMPPA is independent of the ubiquitination of GMPPB

**Figure 5:**
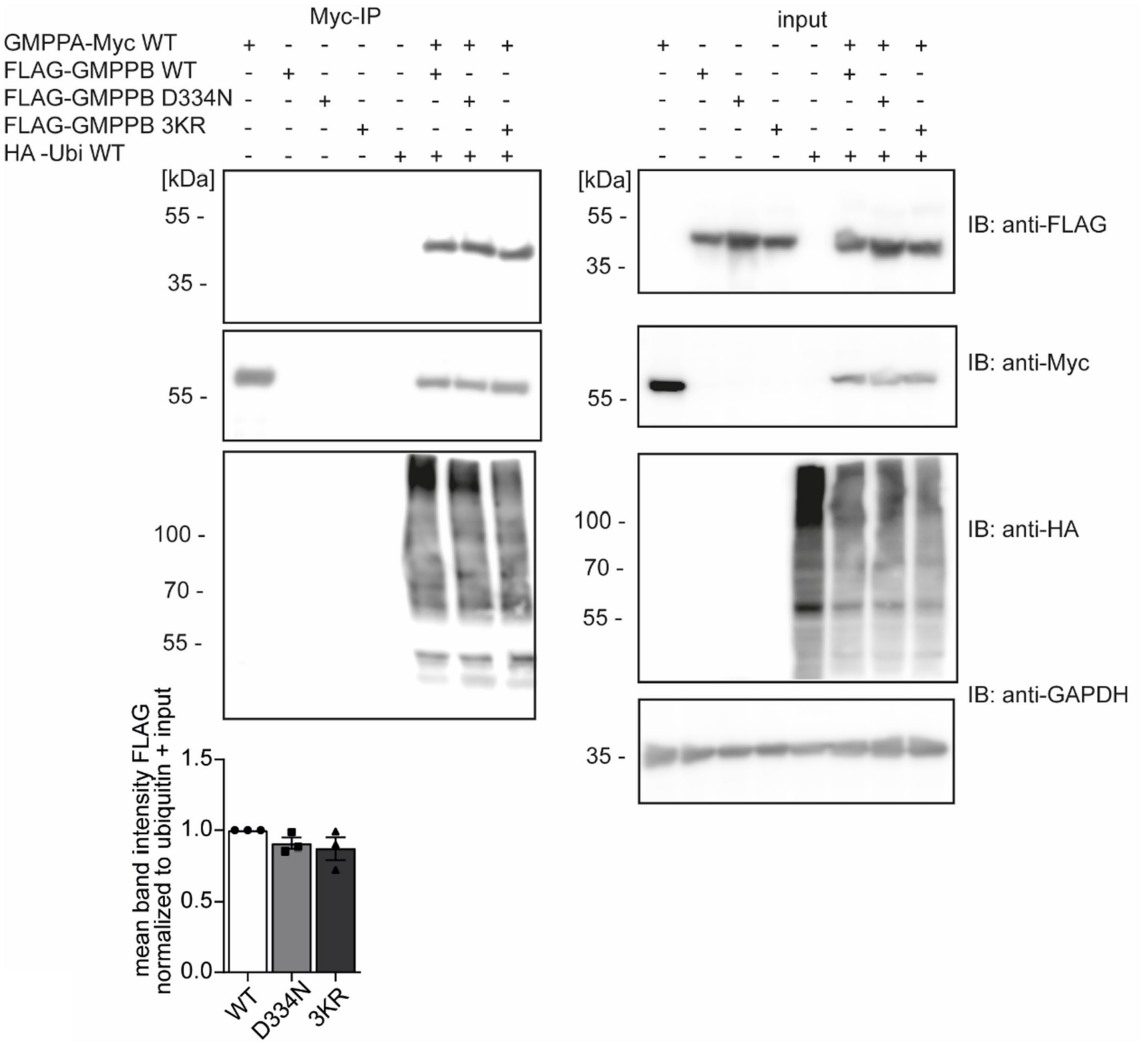
The ubiquitination of GMPPB does not affect its interaction with GMPPA. HEK-293 T are co-transfected with constructs for GMPPA-Myc_6_ together with or without HA-ubiquitin and either FLAG_3_-GMPPB WT, D334N, or GMPPB 3KR, immunoprecipitated via anti-Myc antibodies coupled to beads and analyzed by immunoblot (*n* = 3 experiments, one-way-ANOVA with Fischer’s LSD test). GAPDH is the loading control for input samples. Intensities of pulled-down FLAG-tagged proteins are normalized to the intensity of pulled-down ubiquitinated proteins and FLAG-tagged proteins detected in the input control (normalized to GAPDH). Input boxes represent 5% of the input used for precipitation. Quantitative data are presented as mean ± SEM with individual data points.

Since ubiquitination can influence protein interactions (Foronda et al., 2023), we speculated whether the ubiquitination of GMPPB affects its interaction with GMPPA. To this end, we overexpressed FLAG_3_-GMPPB WT and the disease-associated variant D334N, which is less ubiquitinated (Figure 2), and the ubiquitination-deficient mutant FLAG_3_-GMPPB 3KR together with HA-ubiquitin and GMPPA-Myc_6_ in HEK-293 T cells. We did not observe any difference in the interaction between GMPPA and GMPPB WT or mutated GMPPB variants, and co-expression of HA-ubiquitin also did not influence their interaction (Figure 5; Supplementary Figure S2A). Overall, these data suggest that ubiquitination does not affect the interaction between GMPPB and GMPPA.

### The ubiquitination of GMPPB regulates its enzymatic activity

**Figure 6:**
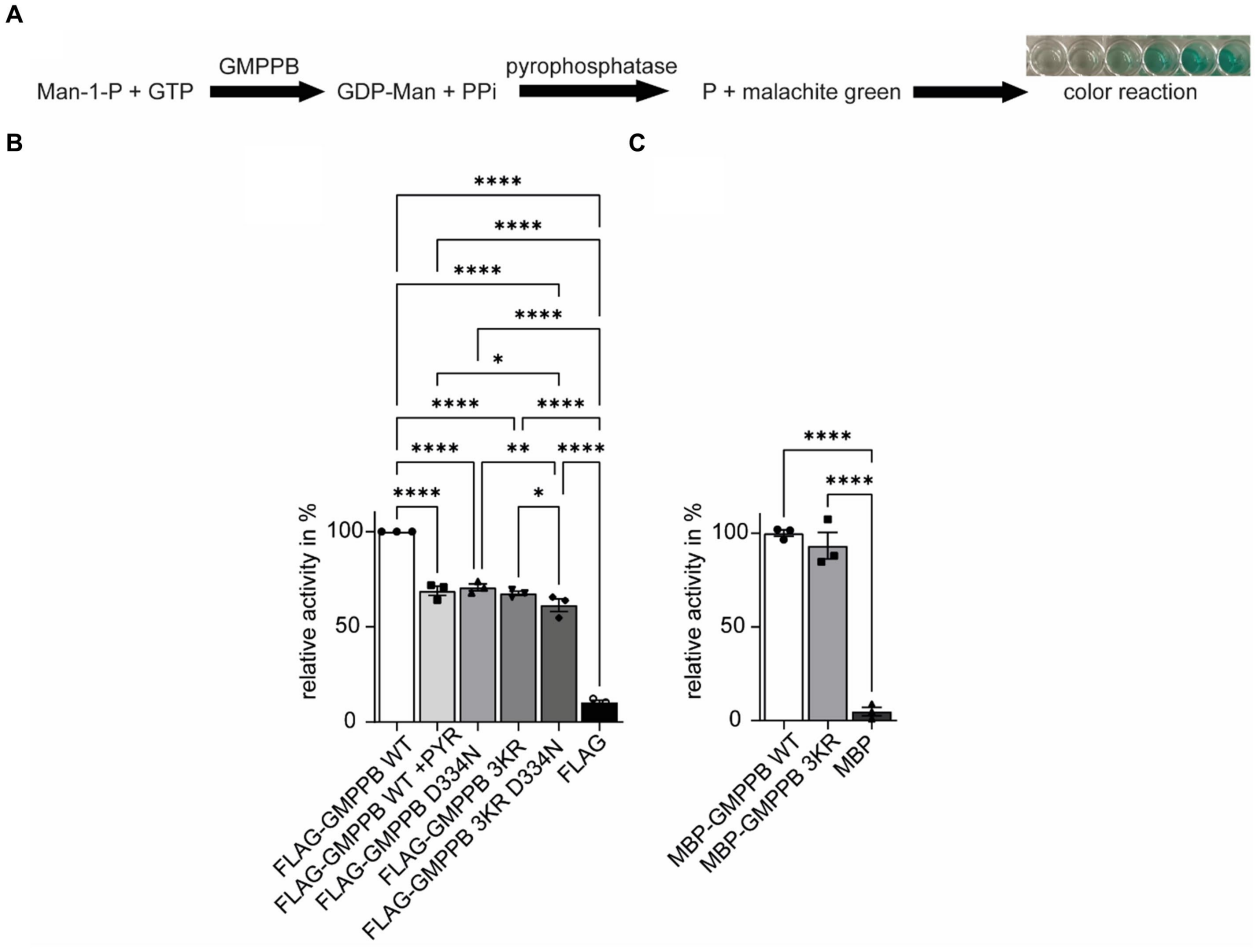
The ubiquitination of GMPPB is important for its enzymatic activity. **(A)** Rationale to monitor the activity of GMPPB. **(B)** Activity measurements were performed in a cuvette format. While FLAG_3_-GMPPB WT displayed enzymatic activity, the activity is significantly reduced for FLAG_3_-GMPPB D334N, FLAG_3_-GMPPB 3KR, and FLAG_3_-GMPPB 3KR D334N. As a control, the ubiquitination of FLAG_3_-GMPPB WT is inhibited with PYR-41 before measuring enzymatic activity. Purified proteins from cells transfected with the empty FLAG_3_-vector alone served as a negative control. Lysate of empty vector-transfected cells served as a negative control (*n* = 3 experiments, one-way-ANOVA with Fischer’s LSD test). **(C)** Activity measurements using recombinant unubiquitinated MBP-GMPPB WT and MBP-GMPPB 3KR. Quantitative data are presented as mean ± SEM with individual data points. *p < 0.05; **p < 0.01; ***p < 0.001; ****p < 0.0001.

In addition to protein turnover and degradation, localization, protein interaction, trafficking, and secretion, ubiquitination can also affect the activity of enzymes (Pickart and Eddins, 2004; Yang et al., 2021). Therefore, we speculated whether this also applies to GMPPB. To this end, we either overexpressed FLAG3-GMPPB WT or the disease-associated D334N variant, the ubiquitination-deficient variant GMPPB 3KR or the 3KR D334N double mutant in HEK-293T cells. After protein enrichment by methanol–chloroform precipitation, we measured the enzymatic activity by colorimetric read-out of generated phosphate in the presence of mannose-1-phosphate, GDP-mannose, GTP, and pyrophosphatase (Figure 6A). Of note, lysine 162 has been reported to be a part of the catalytic center of GMPPB (Zheng et al., 2021). As a control, we also incubated cells expressing FLAG_3_-GMPPB WT with PYR-41, which irreversibly blocks E1 ligases and thus ubiquitination (Yang et al., 2007). Notably, GMPPB activity was significantly reduced for GMPPB D334N, for GMPPB 3KR, and GMPPB 3KR D334N compared to WT (Figure 6B; Supplementary Figures S2B,C). The GMPPB 3KR D334N variant showed a slightly more decreased GMPPB activity compared to the single mutants (Figure 6B; Supplementary Figure S3B). The incubation of the WT control with the ubiquitination blocking agent PYR-41 significantly decreased the enzymatic activity of GMPPB WT (Figure 6B; Supplementary Figure S3B). To exclude that the amino acid substitutions in the GMPPB 3KR mutant might affect the overall structure or arrangement of its active center without ubiquitination being involved, we generated a recombinant GMPPB WT and GMPPB 3KR mutant and measured their enzymatic activity. Notably, we did not detect differences between WT and mutant, suggesting that the amino acid substitutions did not change the arrangement of the catalytic center (Figure 6C; Supplementary Figure S3C).

In summary, the ubiquitination of GMPPB affects its enzymatic activity.

## Discussion

We and others have previously shown that GMPPB, which facilitates the conversion of mannose-1-phosphate and GTP to GDP-mannose (Ning and Elbein, 2000), interacts with its paralogue GMPPA (Zheng et al., 2021; Franzka et al., 2021a). GMPPA lacks enzymatic activity but can still bind GDP-mannose and thus provides a feedback mechanism to limit GMPPB activity as an allosteric feedback inhibitor. Mutations in GMPPB are associated with variable disorders such as muscular dystrophy and other neurological symptoms, including intellectual disability, epilepsy, and cerebellar hypoplasia (Carss et al., 2013; Belaya et al., 2015; Liu et al., 2021).

According to the PhosphoSitePlus database (Hornbeck et al., 2015; Akimov et al., 2018), GMPPB is predicted to be ubiquitinated. In agreement with this prediction, we here show that GMPPB can be precipitated with Ni-NTA beads, which bind to His_6_-ubiquitin. Notably, we observed a second and a faint third band of higher molecular weight, likely representing mono- and poly/multi-ubiquitinated GMPPB proteins. Because these bands were absent from input samples, we hypothesize that they are only detected upon enrichment for ubiquitinated proteins. This suggests that only a fraction of GMPPB is modified by ubiquitination. The lower molecular weight band showed a more prominent intensity compared to the higher molecular weight bands. Possibly, GMPPB forms oligomeric complexes (Zheng et al., 2021) with GMPPA, and thus, potential ubiquitination sites might be covered by this interaction. Upon FLAG_3_-GMPPB co-immunoprecipitation with HA-ubiquitin, we detected several bands at different molecular heights for HA-ubiquitin. These bands might represent mono- and poly/multi-ubiquitinated GMPPB or ubiquitinated interaction partners of GMPPB.

Protein ubiquitination plays a crucial role in various biological processes, including development, cancer (Shi and Grossman, 2010), aging (Hughes et al., 2022), neuronal differentiation and survival (Bax et al., 2019), and synaptic function (Hegde, 2010; Mabb and Ehlers, 2010), as well as axonal guidance and cognitive function (Pinto et al., 2021). Interestingly, ubiquitination is in crosstalk with various other post-translational modifications, including SUMOylation, phosphorylation, acetylation, methylation, hydroxylation, prolyl isomerization, PARylation, neddylation, and *O*-GlcNAcylation (Guan et al., 2018; Barbour et al., 2023). Moreover, ubiquitination can be modulated by *O*-GlcNAcylation (Guinez et al., 2008). In addition to ubiquitination, GMPPB has been predicted to be phosphorylated, methylated, and *O*-GlcNAcylated (Hornbeck et al., 2015), but the functional consequences of the PTMs have not been addressed so far.

Substrate specificity of ubiquitination is brought about by different E3 ligases. Because the E3 ligase TRIM67 was found in the interactome of GMPPB (Oughtred et al., 2021; Demirdizen et al., 2023), we assessed whether TRIM67 and GMPPB indeed interact. Our co-immunoprecipitation studies confirm the interaction, suggesting that TRIM67 might serve as an E3 ligase for GMPPB. Indeed, ubiquitinated GMPPB was almost halved upon knockdown of TRIM67, confirming that TRIM67 acts as an E3 ligase for GMPPB. However, this also indicates that TRIM67 is not the only ligase responsible for GMPPB ubiquitination. Notably, GMPPA was also predicted to be ubiquitinated (Hornbeck et al., 2015) and also interacts with TRIM67 (Oughtred et al., 2021; Demirdizen et al., 2023). TRIM67 is involved in cancer progression (Jiang et al., 2020; Demirdizen et al., 2023), neuritogenesis (Yaguchi et al., 2012), and axonal guidance (Boyer et al., 2020), as well as brain development and cognitive function (Boyer et al., 2018). Remarkably, some disease-associated mutations with reduced enzymatic activity compromised the ubiquitination of GMPPB. Possibly, these mutations impair the binding of the E3 ligase to GMPPB. Since the E3 ligaseTRIM28 and the E2 enzyme UBE2V1 have been found to interact with GMPPB (Jang et al., 2018; Oughtred et al., 2021), and TRIM28 and PARK2 have been found in the interactome of GMPPA (Wan et al., 2015; Jang et al., 2018; Oughtred et al., 2021; Sun et al., 2022), both proteins represent further potential E3 ligase candidates contributing to GMPPB ubiquitination, which should be assessed in future studies.

Ubiquitin itself contains seven different lysine residues (K6, K11, K27, K29, K33, K48, and K63) that potentially can be used for ubiquitin modifications (Mallette and Richard, 2012; Callis, 2014). Depending upon the amount of ubiquitin molecules attached to a protein and the lysine linkage, proteins are targeted to different outcomes. Mono-ubiquitination, for example, is involved in DNA repair and gene expression (Passmore and Barford, 2004), while poly-ubiquitination is involved in protein degradation, signal transduction, or kinase activation (Passmore and Barford, 2004). Notably, GMPPB ubiquitination was increased for a modified ubiquitin, with lysine 48 being replaced by arginine. This suggests that ubiquitination of GMPPB stabilizes the protein at conditions where GMPPB is not predominantly poly-ubiquitinated at K48.

Ubiquitination often targets proteins for proteasomal degradation via K48-linked poly-ubiquitination (Kaiser and Huang, 2005; Kirkpatrick et al., 2006). Because the abundance of GMPPB did not increase upon the inhibition of proteasomal degradation, we also considered that the ubiquitination may target GMPPB for lysosomal degradation (Marques et al., 2004; Clague and Urbé, 2010). This assumption was confirmed by increased GMPPB levels upon the inhibition of lysosomal degradation with bafilomycin A1 and was further verified by the co-localization of GMPPB with autophagic vesicles, which strongly increased upon the inhibition of autophagy as seen in immunofluorescence and immunoblot analysis. In agreement, a previous study showed that disease-associated GMPPB variants were degraded via autophagy (Tian et al., 2019). Notably, the protein abundance of a GMPPB mutant devoid of all three reported ubiquitin sites still increased upon inhibition of lysosomal degradation. This finding suggests that GMPPB degradation is independent of its ubiquitination. Of note, other proteins necessary for protein mannosylation, such as mannose-phosphate isomerase (MPI) or phospho-mannomutase (PMM), are predicted to be ubiquitinated as well in the PhosphoSitePlus database (Hornbeck et al., 2015). In contrast to GMPPB, however, PMM is degraded by the proteasomal pathway (Vilas et al., 2020). Whether other mannosylation-associated enzymes, such as MPI, are degraded via the proteasomal or lysosomal pathway is still elusive.

Ubiquitin can act as a reversible and dynamic platform for protein–protein interactions. Proteins that contain ubiquitin-binding domains can interact with each other by using ubiquitin as a linker molecule (Magits and Sablina, 2022). Since both GMPPB and GMPPA have been reported to be ubiquitinated (Hornbeck et al., 2015), we speculated whether the ubiquitination of GMPPB is important for its interaction with GMPPA. However, disruption of the three reported ubiquitination sites in GMPPB did not compromise the interaction between GMPPB and GMPPA. Upon HA-ubiquitin and FLAG_3_-GMPPB co-immunoprecipitation with GMPPA-Myc_6_, we detected several bands at different molecular heights for HA-ubiquitin. These bands might represent mono- and poly/multi-ubiquitinated GMPPA or ubiquitinated interaction partners of GMPPA. To date, no other ubiquitinated interaction partners are known for GMPPA besides GMPPB, and it remains elusive whether the ubiquitination of GMPPA might be important for its interaction with GMPPB.

It has been shown that ubiquitination may also affect the activity of transcription factors and signaling proteins (Kim et al., 2003; Zhu et al., 2021). Thus, we considered whether the loss of GMPPB ubiquitination may affect its enzymatic activity. Indeed, blocking protein ubiquitination by irreversible inhibition of E1 ligases with PYR-41 or replacing the three potential ubiquitinated lysines of GMPPB with arginine decreased its catalytic activity. The disease-associated patient variant GMPPB D334N, which is less ubiquitinated, also displayed reduced enzymatic activity, which is in agreement with a recent study (Liu et al., 2021), but it was assumed that this may also be related to a different subcellular localization of the mutant protein (Carss et al., 2013). Interestingly, several enzymes important for protein mannosylation, such as phospho-mannomutase (PMM), mannose-6-phosphate-isomerase (MPI), or protein O-linked mannose *N*-acetylglucosaminyltransferase 1 (POMGNT1), are predicted to be ubiquitinated as well (Hornbeck et al., 2015) and also interact with the E3 ligase TRIM67 (Oughtred et al., 2021; Demirdizen et al., 2023), suggesting that ubiquitination might be common feature of enzymes in the mannosylation pathway. However, this has not been addressed experimentally so far.

In summary, our study shows that ubiquitination of GMPPB does affect neither its stability nor its interaction with GMPPA but modulates its enzymatic activity. Thus, ubiquitination provides another level to regulate GMPPB activity and mannosylation.

## Data availability statement

The original contributions presented in the study are included in the article/Supplementary material, further inquiries can be directed to the corresponding authors.

## Author contributions

PF: Conceptualization, Funding acquisition, Investigation, Methodology, Project administration, Software, Writing – original draft, Writing – review & editing. SM: Conceptualization, Investigation, Methodology, Writing – original draft, Writing – review & editing. AC: Formal analysis, Visualization, Writing – review & editing. OH: Conceptualization, Supervision, Writing – original draft, Writing – review & editing. CH: Conceptualization, Funding acquisition, Project administration, Supervision, Writing – original draft, Writing – review & editing.
