# Supplementary material for: Ubiquitination contributes to the regulation of GDP-mannose pyrophosphorylase B activity
Source: Front Mol Neurosci. 2024 Jun 24;17:1375297. doi: 10.3389/fnmol.2024.1375297 (PMC11228364; doi:10.3389/fnmol.2024.1375297)
Supplement: Supplementary file 1 [file Data_Sheet_1.pdf]

Supplemental Material

Ubiquitination contributes to the regulation of GDP-mannose pyrophosphorylase B activity

Patricia Franzka[1*], Sonnhild Mittag[2], Abhijnan Chakraborty[1], Otmar Huber[2], Christian A Hübner[1*]

1 Institute of Human Genetics, University Hospital Jena, Friedrich Schiller University, Jena, Germany.
2 Department of Biochemistry II, University Hospital Jena, Friedrich Schiller University, Jena, Germany.

*** Correspondence:**
Corresponding Author
Patricia.Franzka@med.uni-jena.de
Christian.Huebner@med.uni-jena.de

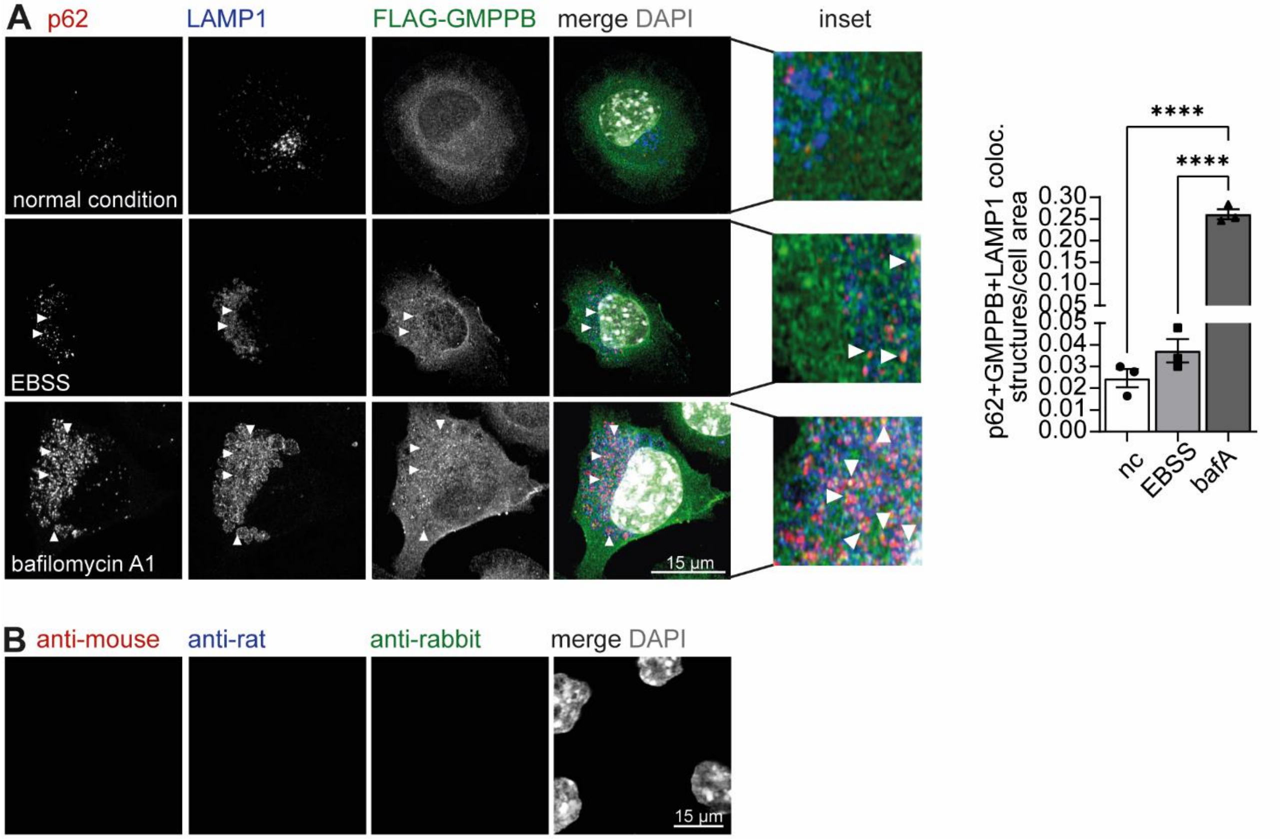

Supplementary Figure 1. GMPPB protein levels are regulated by autophagy. A) EBSS starvation for 6 h or inhibition of lysosomal degradation with bafilomycin A1 increases the co-localization of overexpressed FLAG$_3$-GMPPB with autolysosomes (LAMP1- and p62-positive puncta) in N2A cells. Co-localization was analyzed with the Comdet v05 plugin from ImageJ. N = 3 experiments with 5-10 cells/genotype per condition and experiment, scale bar: 15 µm. White arrowheads indicate exemplary co-localization. Quantitative data are presented as mean ± SEM with individual data points. ****P < 0.0001. B) Antibody control for Figure 4C: Immunofluorescence stainings with anti-mouse-coupled

Cy5 Alexa Fluorophore, anti-rat-coupled 555 Alexa Fluorophore anti-rabbit-coupled 488 Alexa Fluorophore and DAPI (nuclei) (scale bar: 15 µm).

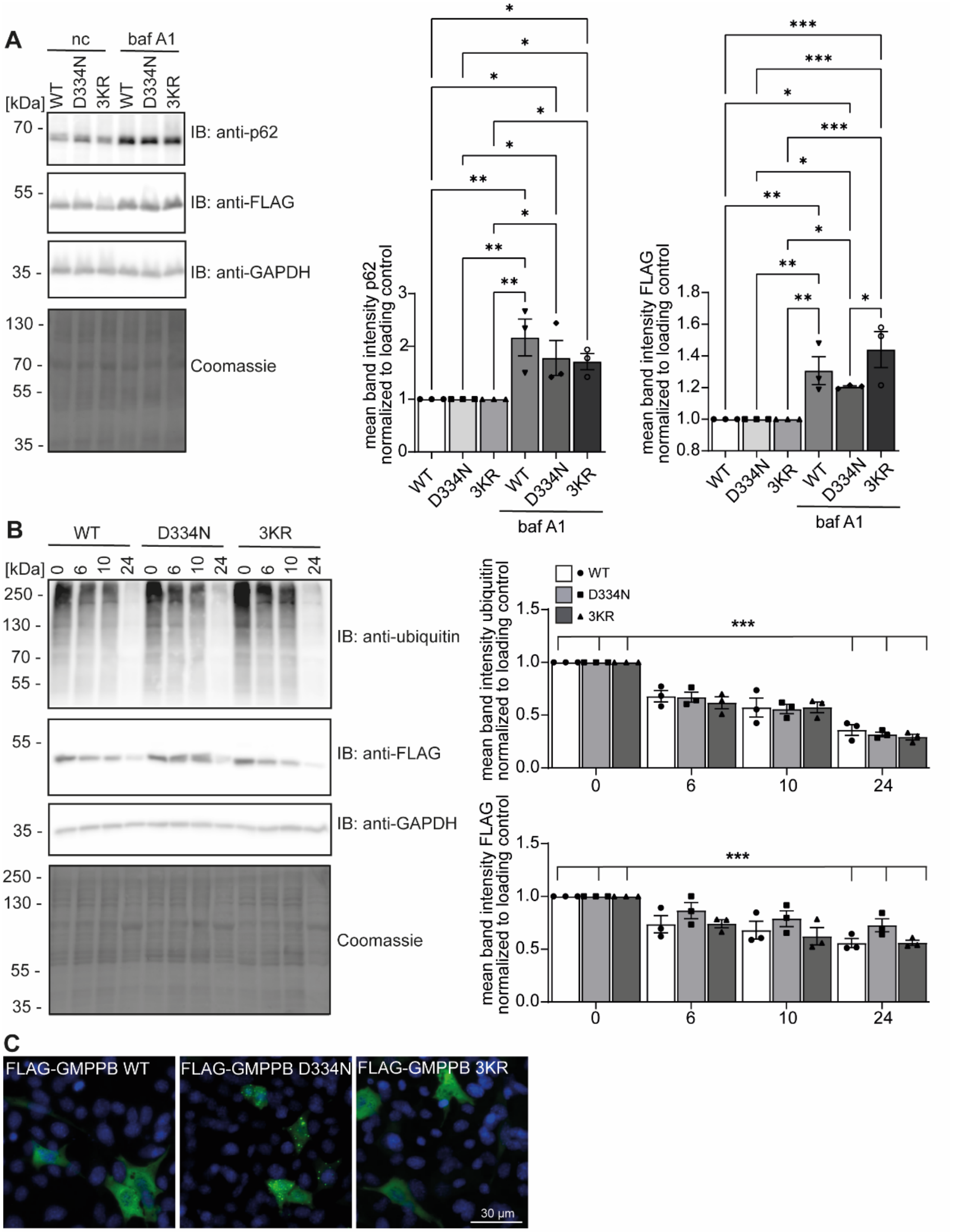

Supplementary Figure 2. Ubiquitination of GMPPB is not important for its degradation. A) HEK-293T cells were transfected with $FLAG_3$-GMPPB WT, D334N or 3KR mutant constructs. Incubation with bafilomycin A1 resulted in increased $FLAG_3$-GMPPB levels. P62 served as a control for efficient bafilomycin A1 treatment. GAPDH and Coomassie staining served as loading control. Mean band intensities were normalized to GAPDH (n = 3 experiments, 1-way-ANOVA with Fischer´s

LSD test). **B)** HEK-293T cells were transfected with $FLAG_3$-GMPPB WT, D334N or 3KR mutant constructs. Incubation with cycloheximide (CHX) resulted in decreasing GMPPB levels independent of the mutation. Ubiquitin served as a control for efficient CHX treatment. GAPDH and Coomassie staining served as loading control. Mean band intensities were normalized to GAPDH (n = 3 experiments, 1-way-ANOVA with Fischer´s LSD test). **C)** Representative immunofluorescence images of HEK-293T cells transfected with $FLAG_3$-GMPPB WT, D334N or 3KR mutant constructs (scale bar: 30 µm). Quantitative data are presented as mean ± SEM with individual data points. *P < 0.05; **P < 0.01; ***P < 0.001.

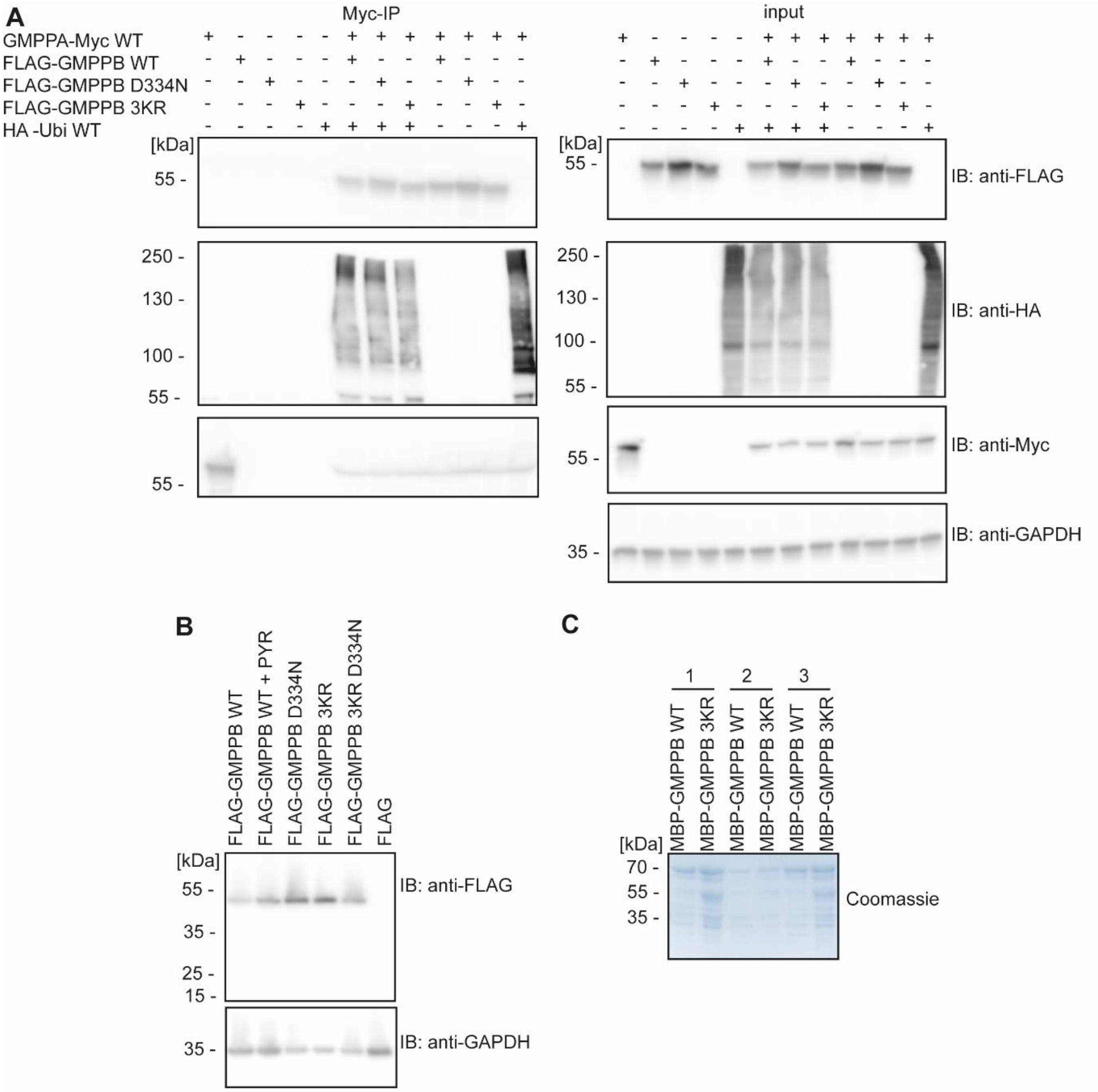

Supplementary Figure 3. Ubiquitination of GMPPB is not needed for its interaction with GMPPA, but for its activity. A) Representative blots of overexpression of $GMPPA-Myc_6$ together with or without HA-ubiquitin and $FLAG_3$-GMPPB WT, D334N and K3R with subsequent anti-Myc-IP. **B)** Immunoblot analysis of overexpressed and enriched protein lysates used for activity assays. GAPDH served as loading control. **C)** Coomassie gels of recombinant MBP-GMPPB WT and MBP

GMPPB 3KR. Numbers (1-3) indicate round of experiment/purification. For enzyme activity assays, protein amount was estimated from this input gel.

Supplementary Figure 4. Full Western Blots/ gels for main and supplementary figures.
