# Supplementary material for: Ubiquitination contributes to the regulation of GDP-mannose pyrophosphorylase B activity
Source: Front Mol Neurosci. 2024 Jun 24;17:1375297. doi: 10.3389/fnmol.2024.1375297 (PMC11228364; doi:10.3389/fnmol.2024.1375297)

Figure 1 A, B

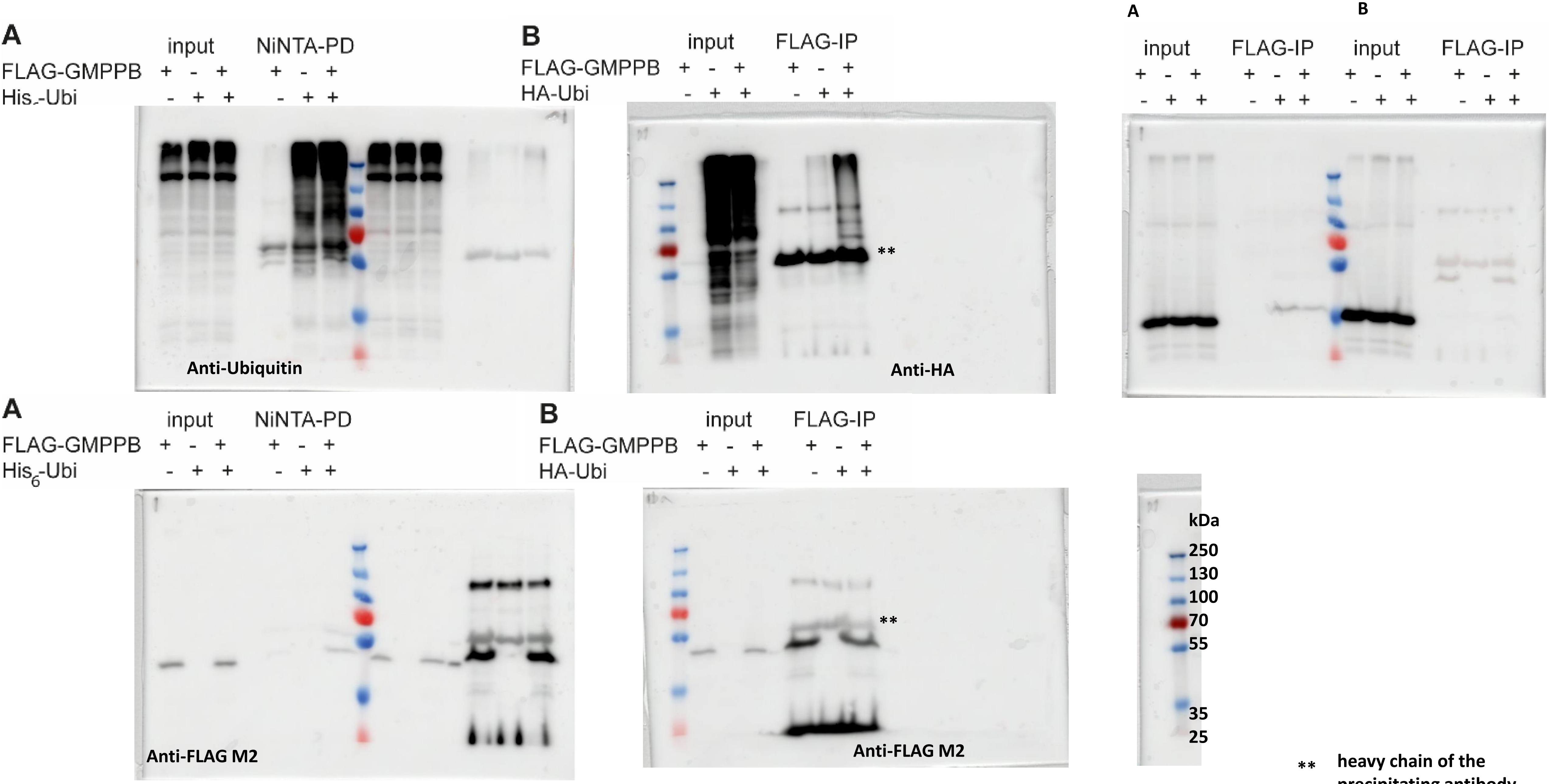

** heavy chain of the precipitating antibody

Figure 1 C

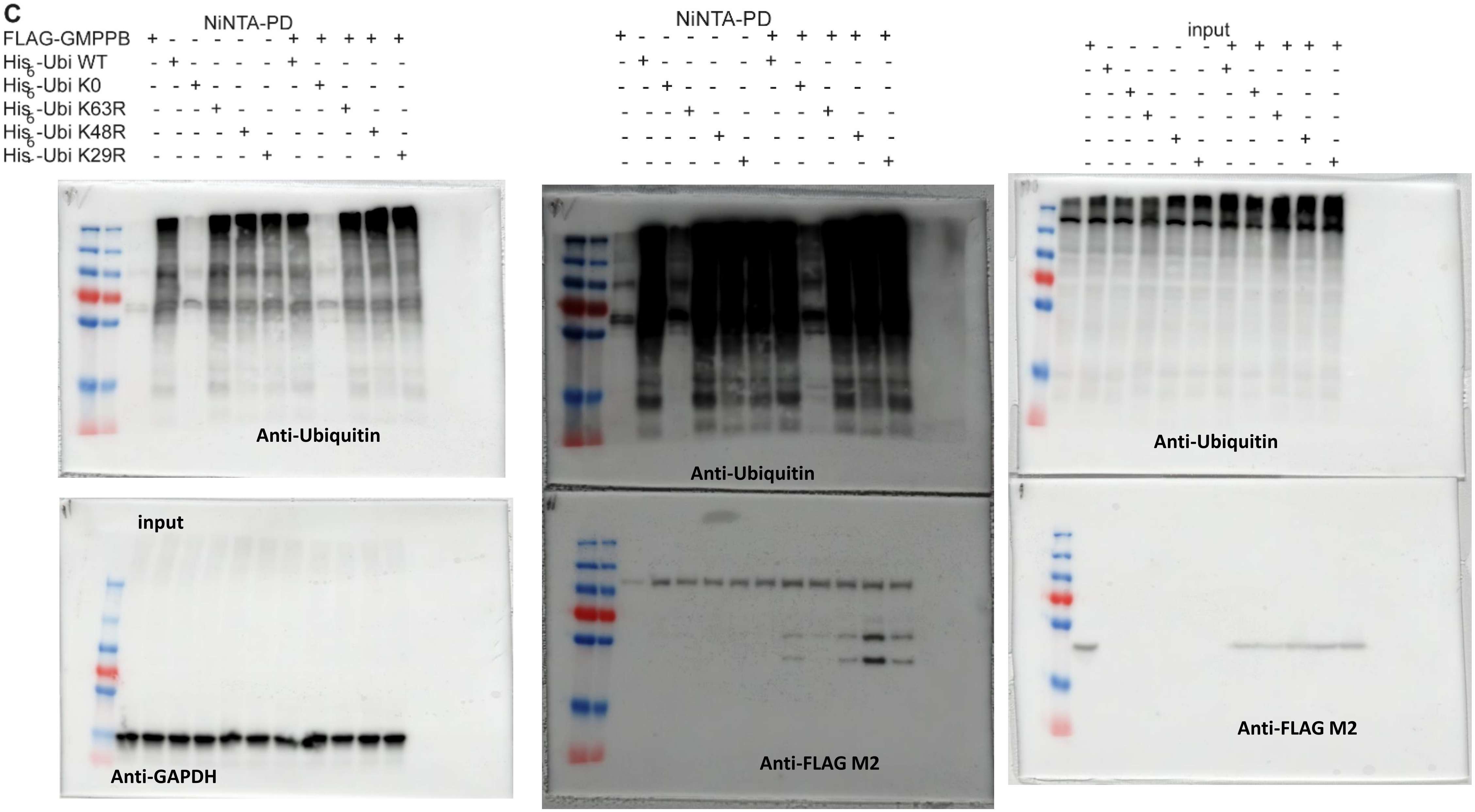

C
NiNTA-PD
FLAG-GMPPB + - - - - - + + + + +
His6-Ubi WT - + - - - - + - - - -
His6-Ubi K0 - - + - - - - + - - -
His6-Ubi K63R - - - + - - - - + - -
His6-Ubi K48R - - - - + - - - - + -
His6-Ubi K29R - - - - - + - - - - +
Anti-Ubiquitin
input
Anti-GAPDH
NiNTA-PD
Anti-Ubiquitin
Anti-FLAG M2
input
Anti-Ubiquitin
Anti-FLAG M2

Figure 2

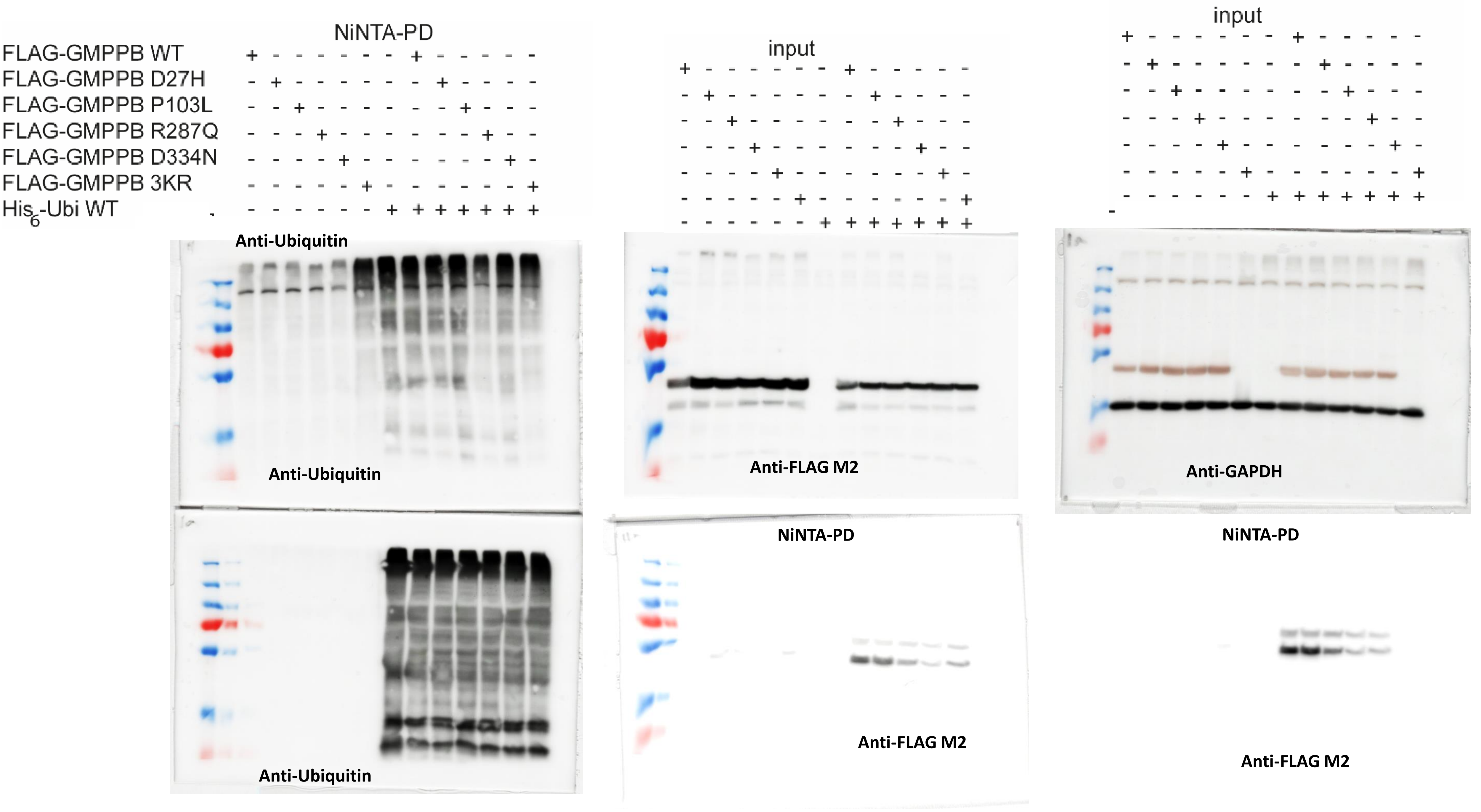
NiNTA-PD
FLAG-GMPPB WT
FLAG-GMPPB D27H
FLAG-GMPPB P103L
FLAG-GMPPB R287Q
FLAG-GMPPB D334N
FLAG-GMPPB 3KR
His6-Ubi WT
input
input
Anti-Ubiquitin
Anti-Ubiquitin
Anti-FLAG M2
Anti-GAPDH
NiNTA-PD
NiNTA-PD
Anti-Ubiquitin
Anti-FLAG M2
Anti-FLAG M2

Figure 3 A

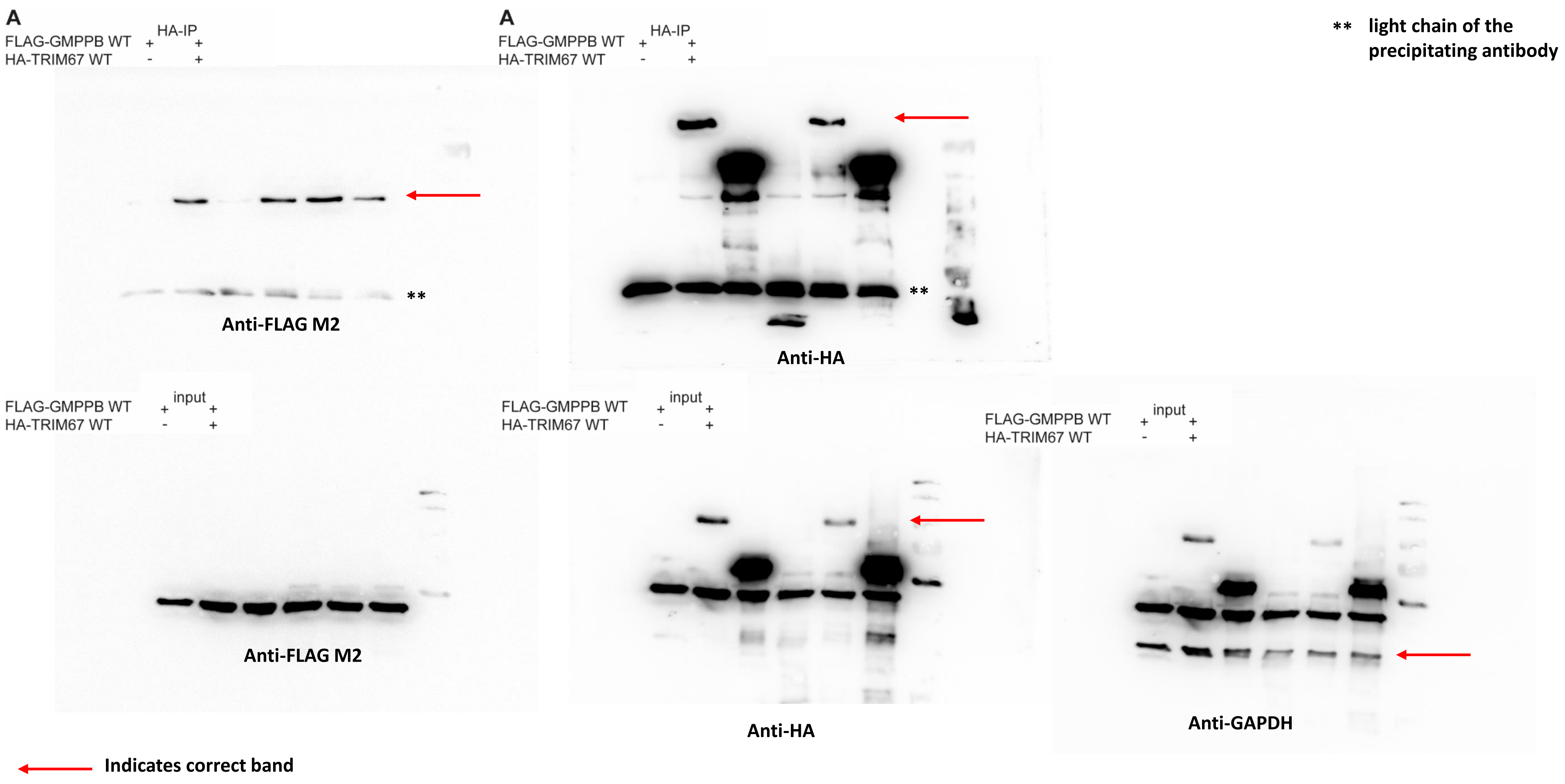

A
FLAG-GMPPB WT + +
HA-TRIM67 WT - +
HA-IP
**
Anti-FLAG M2
A
FLAG-GMPPB WT + +
HA-TRIM67 WT - +
HA-IP
**
Anti-HA
** light chain of the precipitating antibody
FLAG-GMPPB WT + +
HA-TRIM67 WT - +
input
Anti-FLAG M2
FLAG-GMPPB WT + +
HA-TRIM67 WT - +
input
Anti-HA
FLAG-GMPPB WT + +
HA-TRIM67 WT - +
input
Anti-GAPDH
Indicates correct band

Figure 3 B

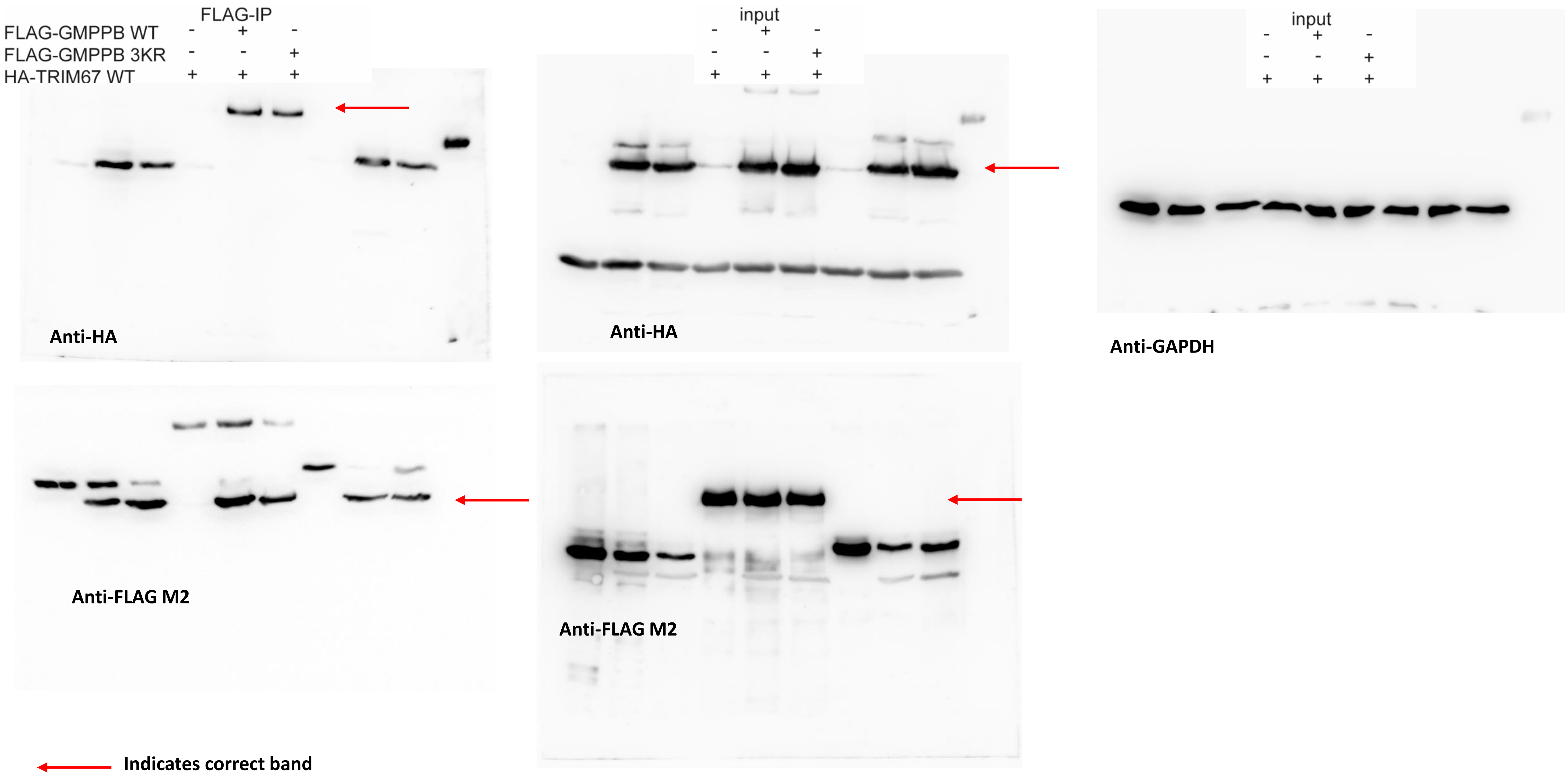
FLAG-IP
FLAG-GMPPB WT - + -
FLAG-GMPPB 3KR - - +
HA-TRIM67 WT + + +
Anti-HA
Anti-FLAG M2
input
- + -
- - +
+ + +
Anti-HA
Anti-FLAG M2
input
- + -
- - +
+ + +
Anti-GAPDH
Indicates correct band

Figure 3 C

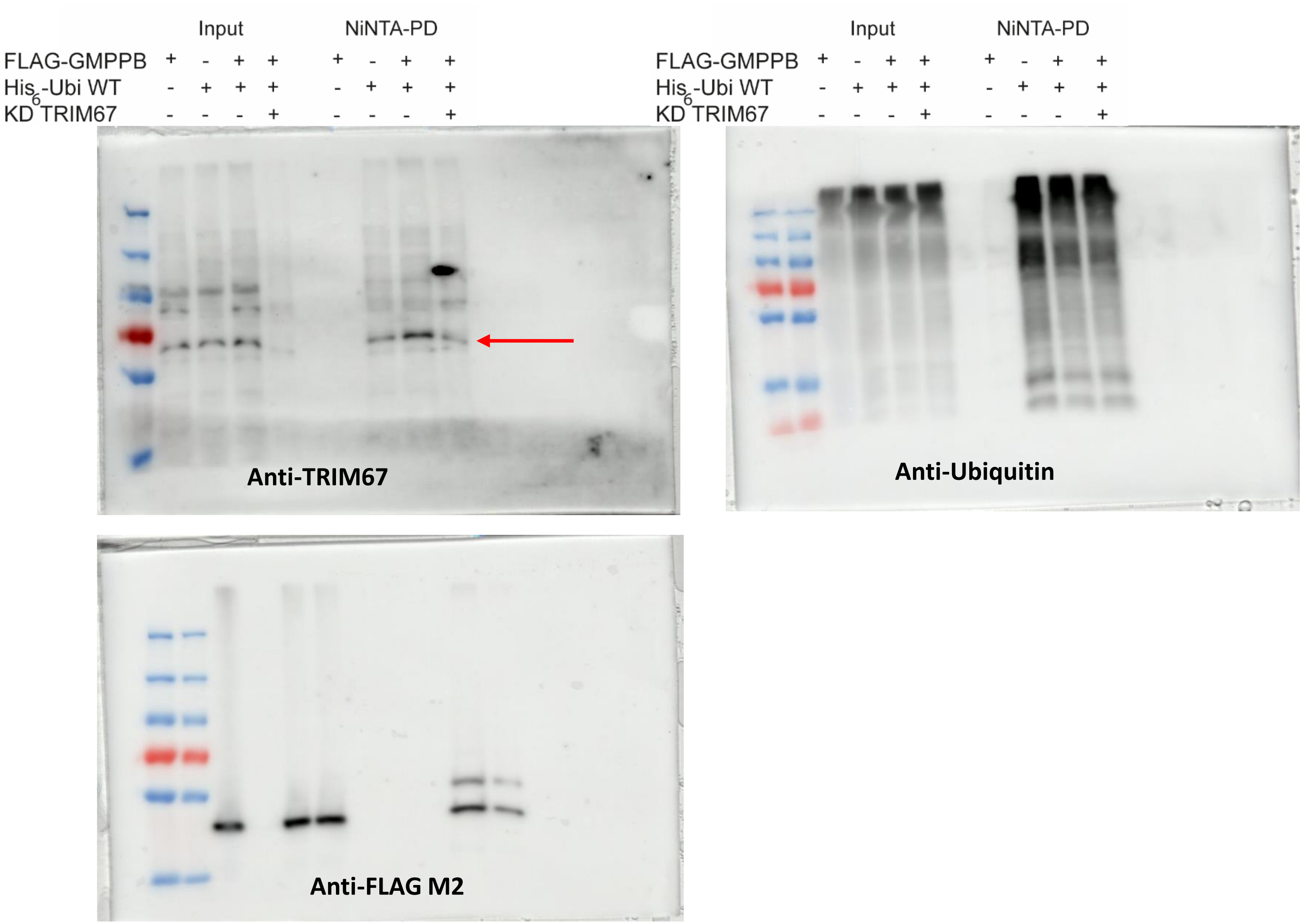

← **Indicates correct band**

Figure 4

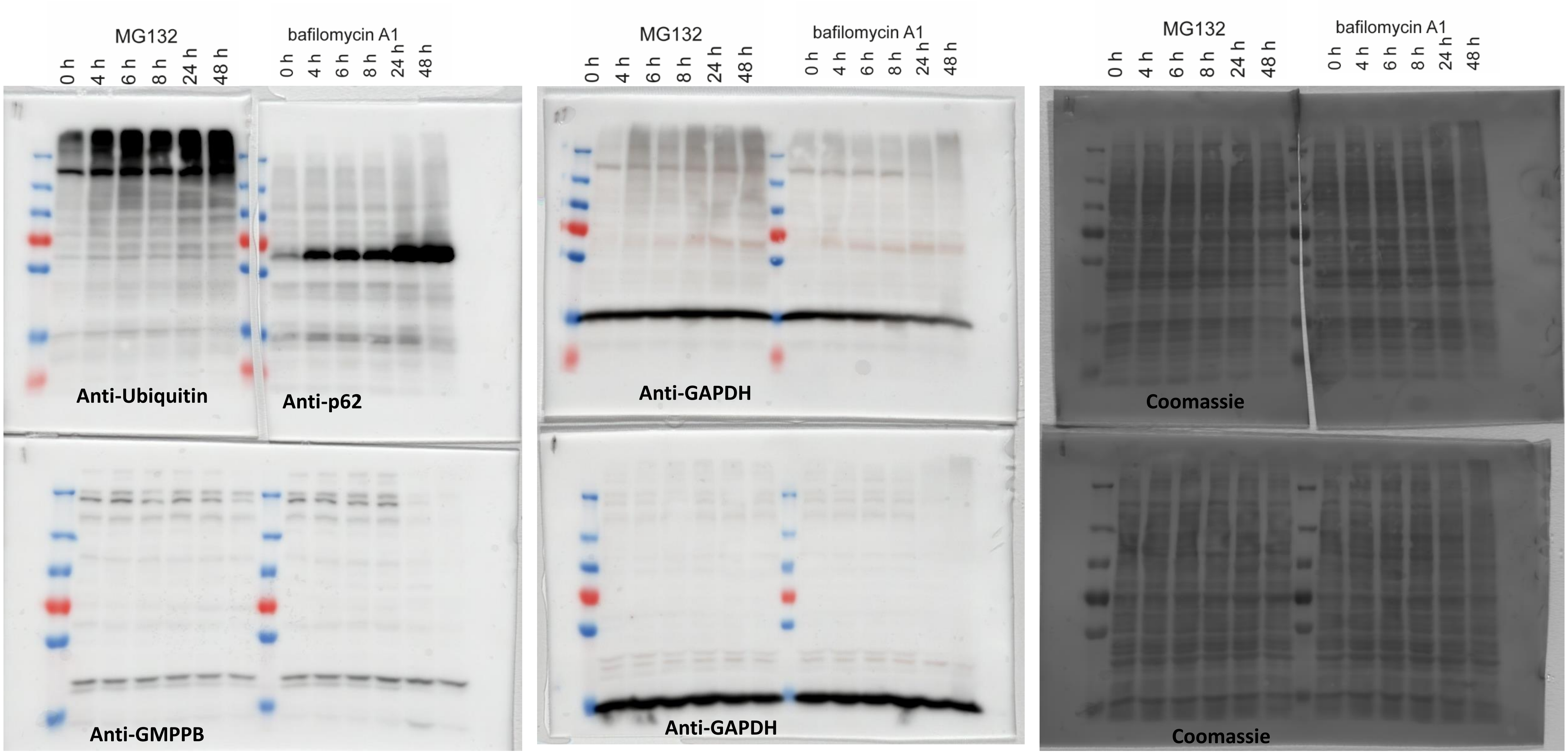

MG132
bafilomycin A1
0 h
4 h
6 h
8 h
24 h
48 h
Anti-Ubiquitin
Anti-p62
Anti-GMPPB
Anti-GAPDH
Anti-GAPDH
Coomassie
Coomassie

Figure 5

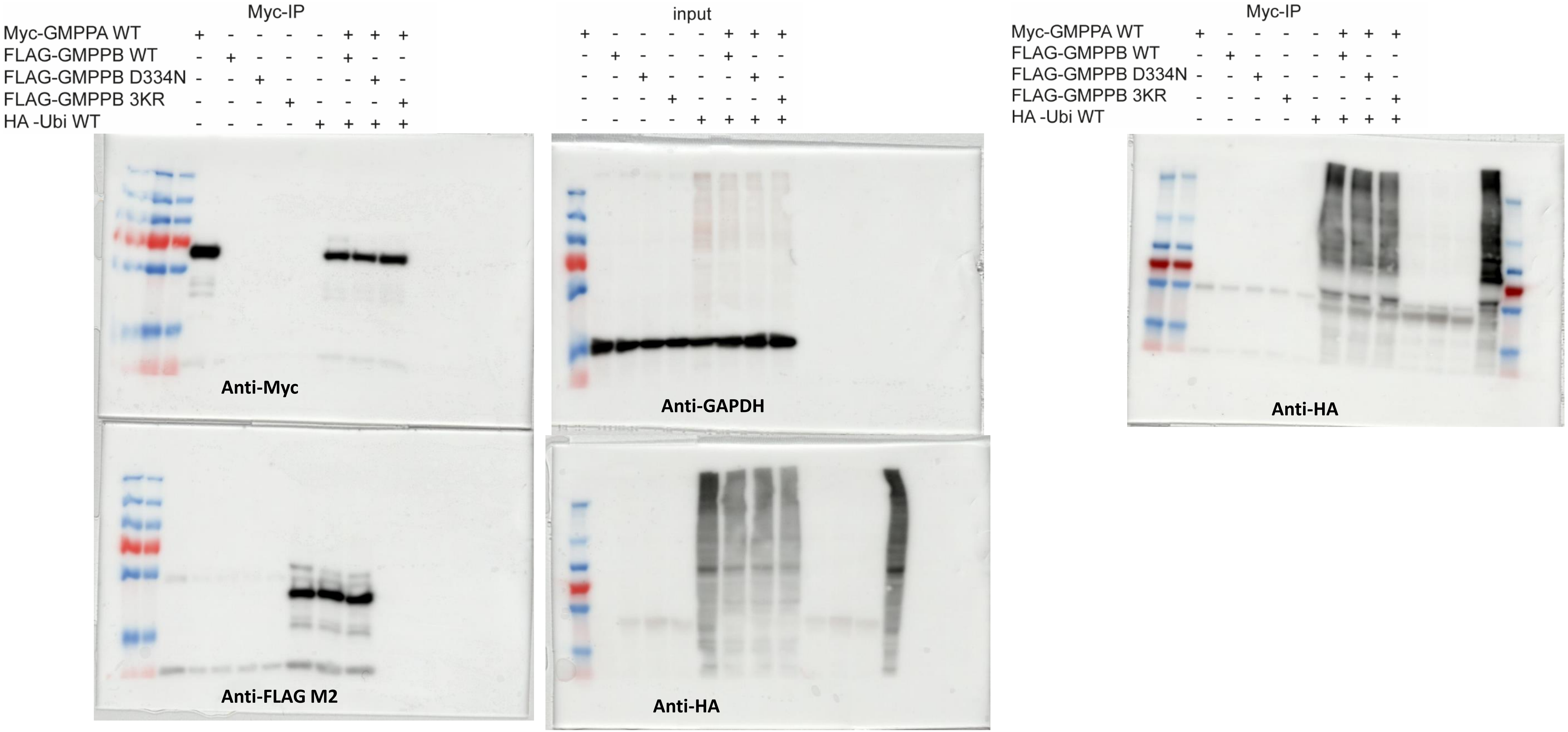
Myc-IP
Myc-GMPPA WT + - - - - + + +
FLAG-GMPPB WT - + - - - + - -
FLAG-GMPPB D334N - - + - - - + -
FLAG-GMPPB 3KR - - - + - - - +
HA -Ubi WT - - - - + + + +
Anti-Myc
Anti-FLAG M2
input
+ - - - - + + +
- + - - - + - -
- - + - - - + -
- - - + - - - +
- - - - + + + +
Anti-GAPDH
Anti-HA
Myc-IP
Myc-GMPPA WT + - - - - + + +
FLAG-GMPPB WT - + - - - + - -
FLAG-GMPPB D334N - - + - - - + -
FLAG-GMPPB 3KR - - - + - - - +
HA -Ubi WT - - - - + + + +
Anti-HA

Supplementary Figure 2 A

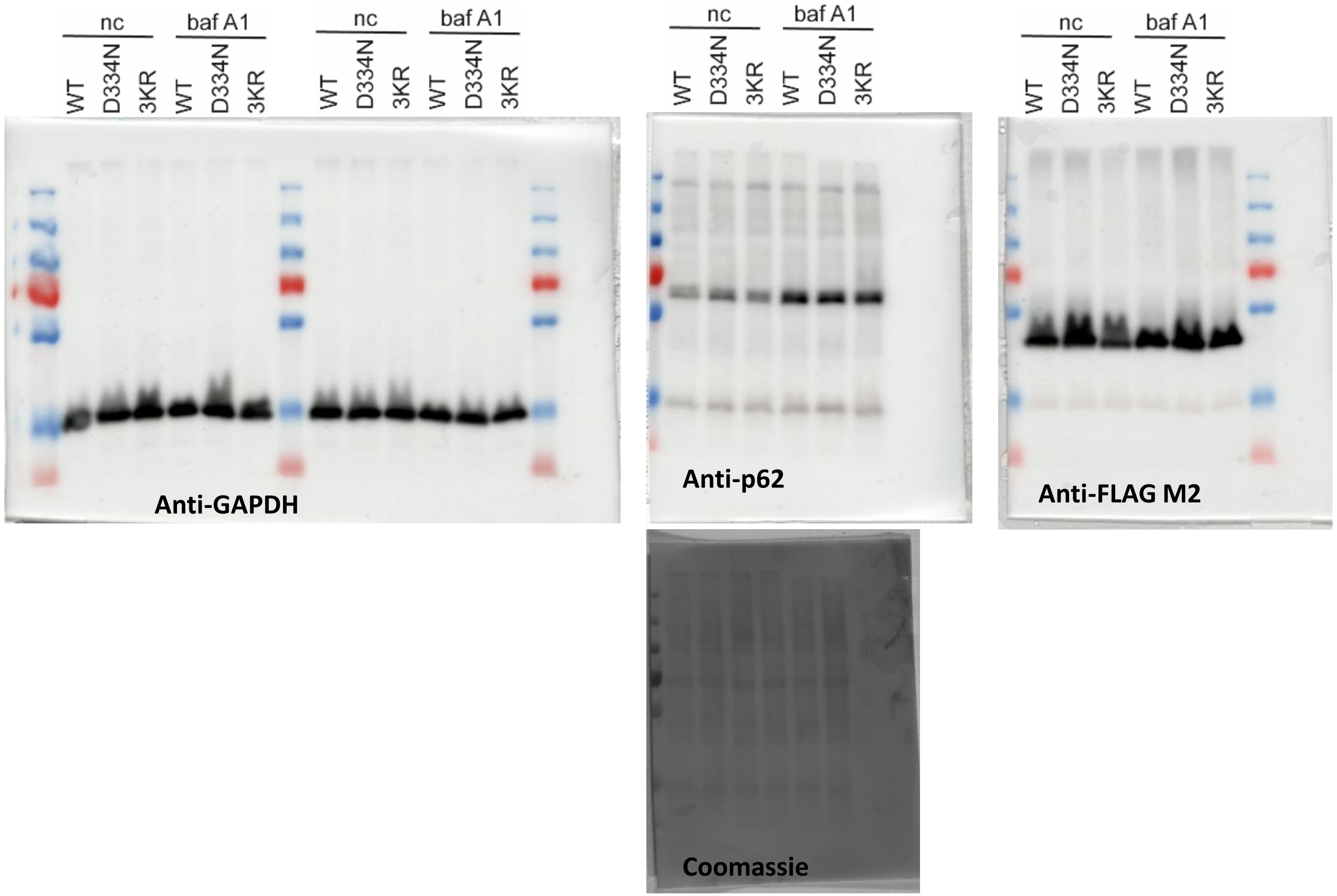

Supplementary Figure 2 B

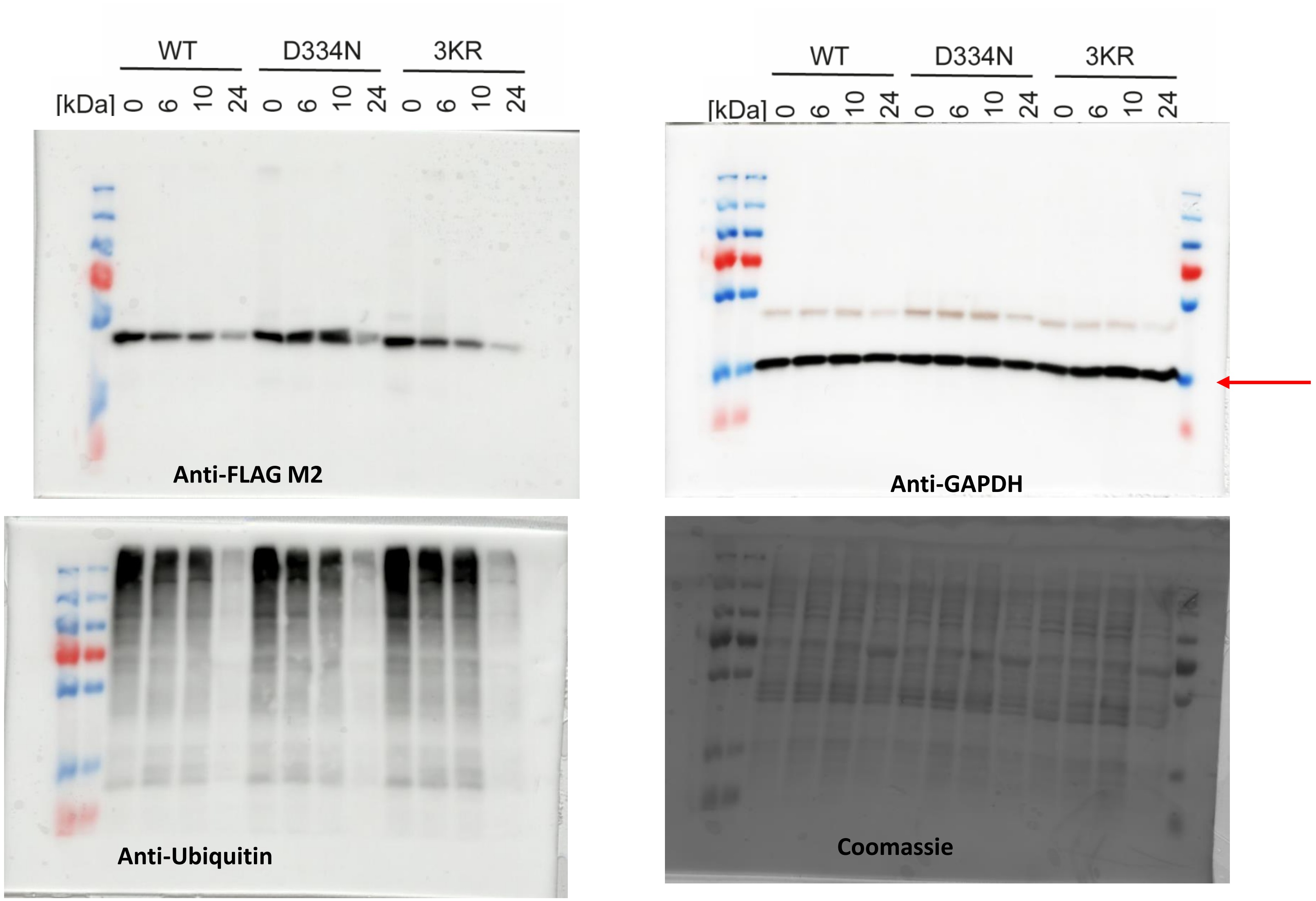

Indicates correct band

Supplementary Figure 3 A

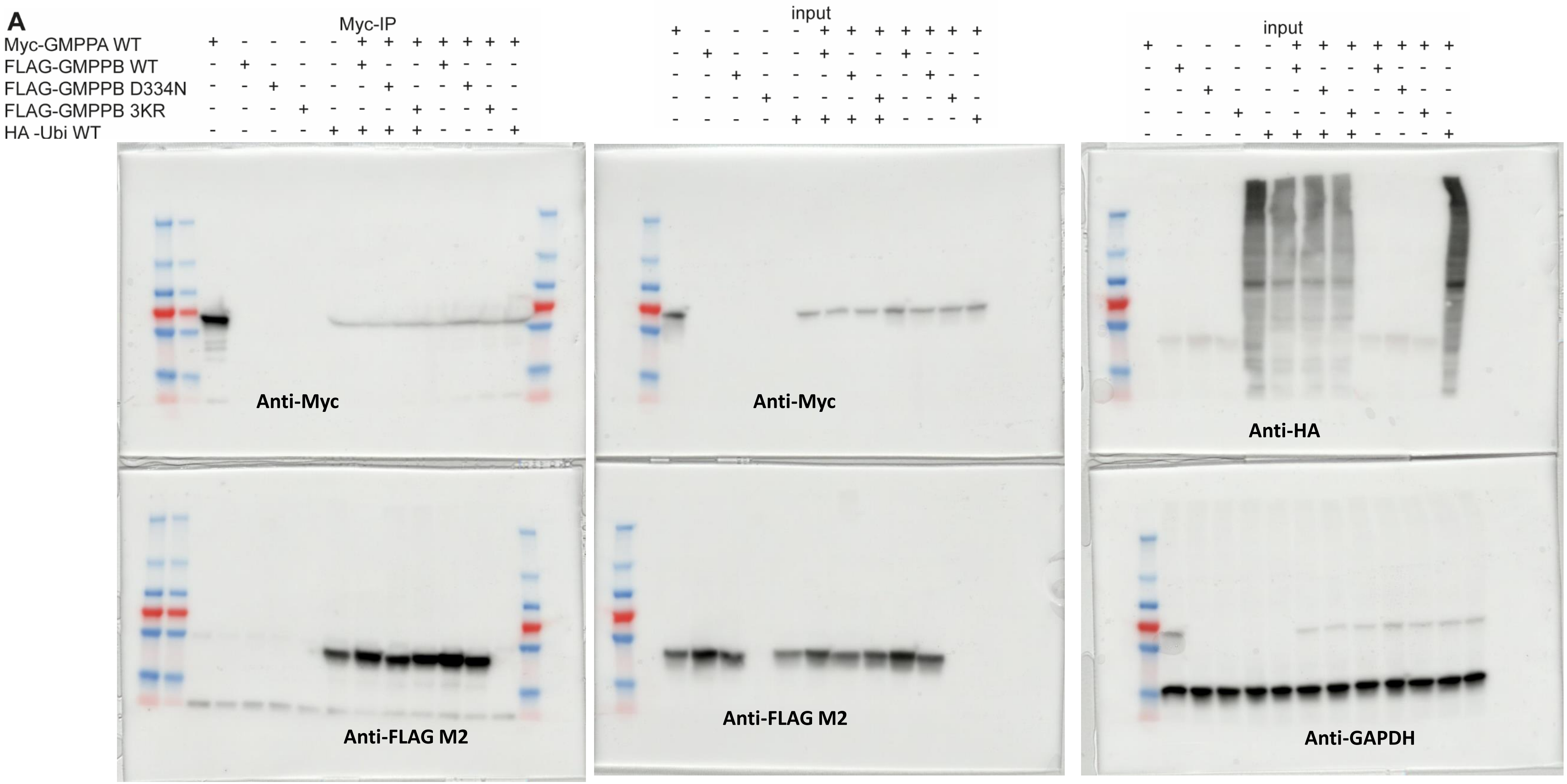

Supplementary Figure 3 A

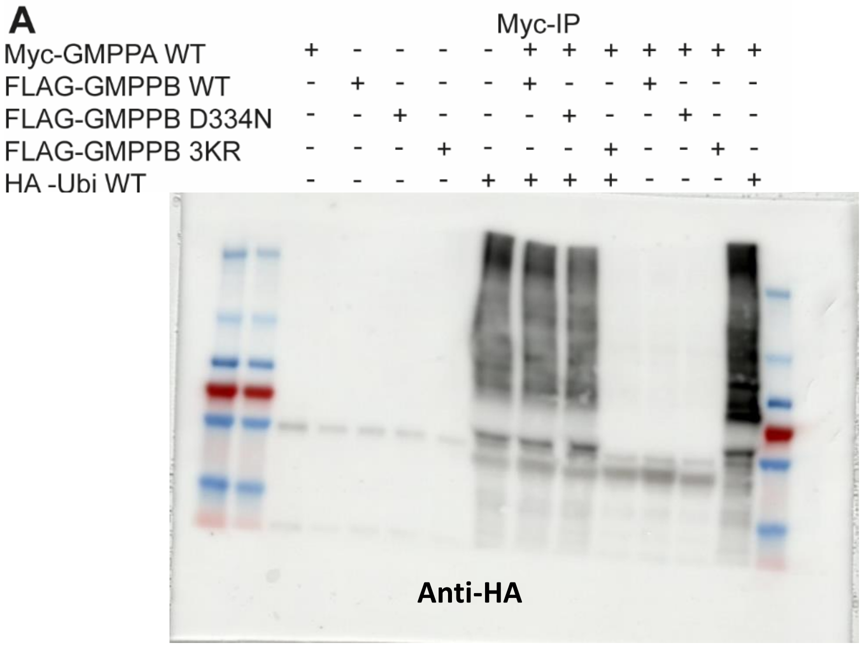

Supplementary Figure 3 B

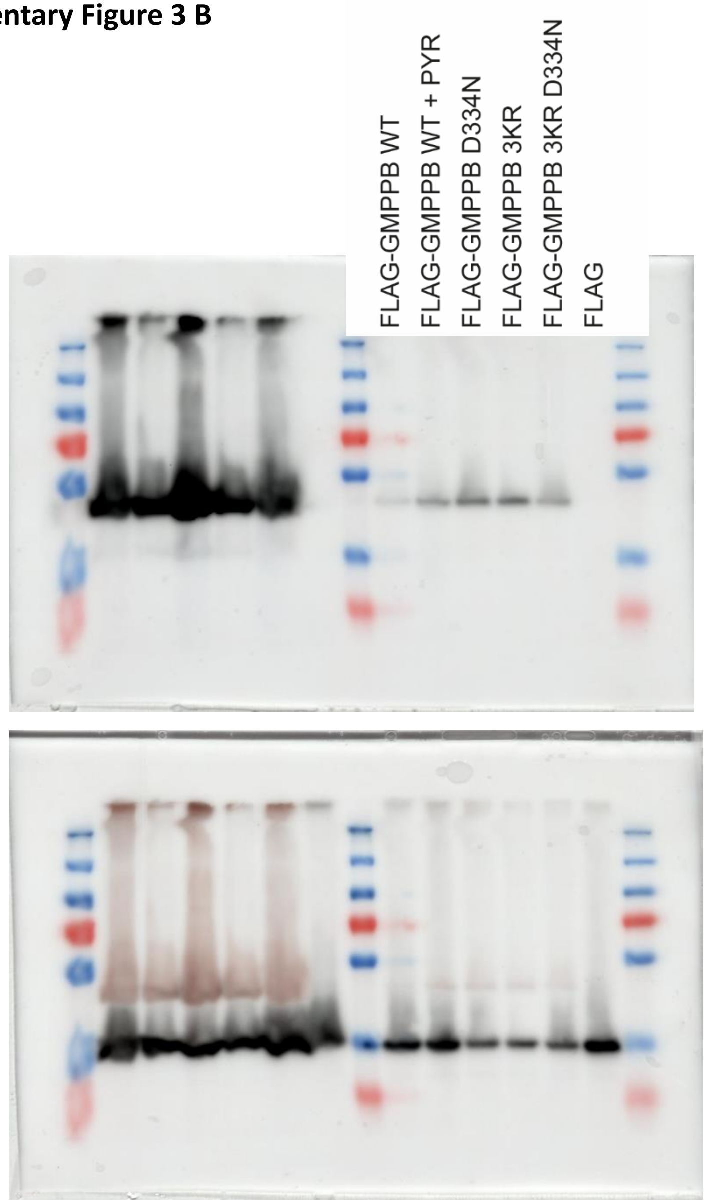

Supplementary Figure 3 C

14.03.2024

1. Aufreinigung Bakterienlysat1 12.03.2024 (1)
2. Aufreinigung Bakterienlysat1 13.03.2024 (2)
3. Aufreinigung Bakterienlysat2 13.03.2024 (3)

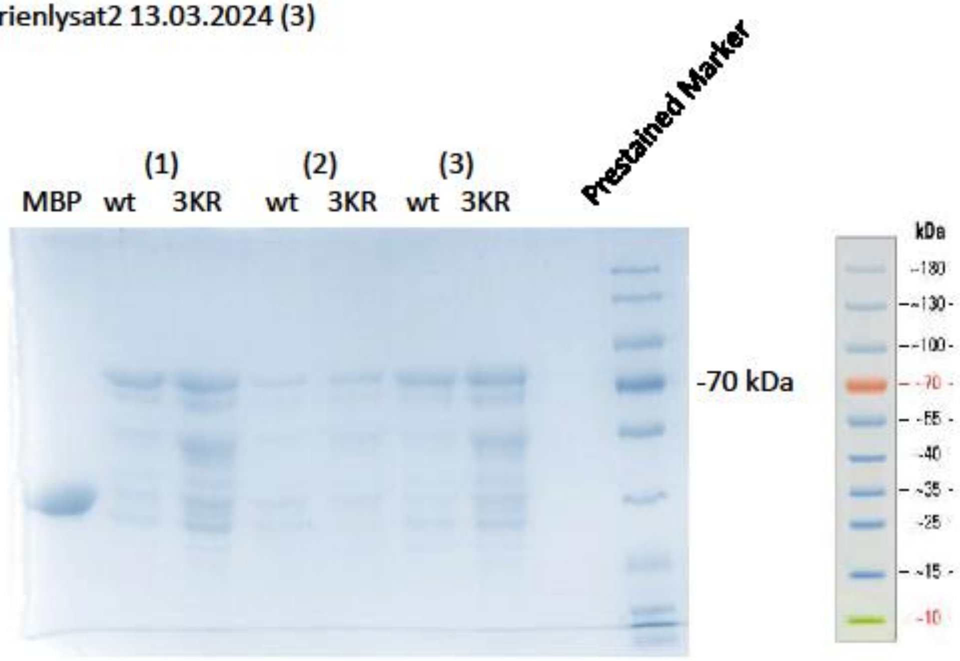

Supplement: Supplementary file 2 [file Data_Sheet_2.pdf]
